# Impact of Poly(Lactic Acid) and Graphene Oxide Nanocomposite on Cellular Viability and Proliferation

**DOI:** 10.3390/pharmaceutics17070892

**Published:** 2025-07-09

**Authors:** Karina Torres Pomini, Júlia Carolina Ferreira, Laira Mireli Dias da Silva, Paulo Gabriel Friedrich Totti, Monique Gonçalves Alves, Eliana de Souza Bastos Mazuqueli Pereira, Marcelo Melo Soares, Durvanei Augusto Maria, Rose Eli Grassi Rici

**Affiliations:** 1 Department of Human Morphophysiology, School of Medicine, University of Marília (UNIMAR), Avenida Hygino Muzzy Filho, 1001, Marília 17525-902, SP, Brazil; 2 Interdisciplinary Master’s Program in Structural and Functional Interactions in Rehabilitation, University of Marília (UNIMAR), Avenida Hygino Muzzy Filho, 1001, Marília 17525-902, SP, Brazil; lairadias@outlook.com.br (L.M.D.d.S.); paulo.curso_@outlook.com (P.G.F.T.); elianabastosmsn@hotmail.com (E.d.S.B.M.P.); 3 Department of Periodontics, University of Marília (UNIMAR), Avenida Hygino Muzzy Filho, 1001, Marília 17525-902, SP, Brazil; jucarolina18@gmail.com; 4 Development and Innovation Laboratory, Butantan Institute, Avenida Vital Brasil, 1500—Butantã, São Paulo 05503-900, SP, Brazil; moniquealves@usp.br (M.G.A.); durvanei.maria@butantan.gov.br (D.A.M.); 5 Graduate Program in Medical Sciences, College of Medicine, University of São Paulo (USP), Rua da Biblioteca, 21, São Paulo 05508-220, SP, Brazil; 6 Department of Prosthodontics, University of Marília (UNIMAR), Avenida Hygino Muzzy Filho, 1001, Marília 17525-902, SP, Brazil; 7 Orofacial Rehabilitation Institute Osteogenesis S/S LTDA, Rua Dr. Guilherme Bannitz, 90, São Paulo 04532-060, SP, Brazil; marcelomelo61@gmail.com; 8 Graduate Program in Anatomy of Domestic and Wild Animals, University of São Paulo (USP), São Paulo 05508-270, SP, Brazil

**Keywords:** tissue engineering, nanocomposite, graphene oxide, biocompatible materials, poly(lactic acid), fibroblasts cells/metabolism, mesenchymal stem cells/metabolism, human umbilical vein endothelial cells/metabolism, biological specimen banks

## Abstract

**Background/Objectives:** Although the nanocomposite of poly(L-lactic acid) with graphene oxide (PLLA-GO) shows promise for tissue engineering, its specific bioactive interactions with diverse cell lineages during early tissue regeneration remain unclear. This study comprehensively investigated the in vitro multifaceted biocompatibility of PLLA-GO using human fibroblasts (FN1 cells), murine mesenchymal stem cells (mBMSCs), and human umbilical vein endothelial cells (HUVECs). **Methods:** Morphological analyses were performed using optical and scanning electron microscopy, while proliferation dynamics were assessed via CFSE staining. Cell cycle progression was evaluated using flow cytometry, mitochondrial activity was examined through TMRE staining, and inflammatory cytokine profiling was performed via Cytometric Bead Array (CBA). **Results:** PLLA-GO exhibited primary biocompatibility across all evaluated cell lines, characterized by efficient adhesion and proliferation. However, significant cell-type-dependent modulations were observed. The FN1 cells exhibited proliferative adaptation but induced accelerated scaffold degradation, as evidenced by a substantial increase in cellular debris (5.93% control vs. 34.38% PLLA-GO; *p* = 0.03). mBMSCs showed a transient initial proliferative response and a significant 21.66% increase in TNF-α production (179.67 pg/mL vs. 147.68 pg/mL in control; *p* = 0.03). HUVECs demonstrated heightened mitochondrial sensitivity, exhibiting a 32.19% reduction in mitochondrial electrical potential (97.07% control vs. 65.82% PLLA-GO; *p* ≤ 0.05), alongside reductions in pro-inflammatory cytokines TNF-α (8.73%) and IL-6 (12.47%). **Conclusions:** The PLLA-GO processing method is crucial for its properties and subsequent cellular interactions. Therefore, rigorous and specific preclinical evaluations—considering both cellular contexts and fabrication—are indispensable to ensure the safety and therapeutic potential of PLLA-GO in tissue engineering and regenerative medicine.

## 1. Introduction

Tissue engineering (TE), a continually evolving multidisciplinary field, is dedicated to the development of biological substitutes with the aim of restoring, repairing, or enhancing the function of damaged or lost tissues and organs [1,2]. In this context, the success of effective regenerative therapies hinges critically on the synergistic interactions among cells, growth factors, and biomimetic scaffolds [3].

The design of scaffold materials that integrate physical and biological characteristics, as well as facilitate the emission of essential biological signals, is fundamental to mimic a specific regenerative niche, promoting cellular development, morphogenesis, and, consequently, the re-establishment of the tissue’s morphofunctional integrity [3,4].

For these biological substitutes to be effective, they must exhibit carefully optimized physical and biological properties. This includes biodegradability, with a degradation rate synchronous with new tissue growth, and the ability to mimic the appropriate mechanical properties within an in vivo environment, resisting physiological forces [5,6].

Among the biocompatible polymers, poly-L-lactic acid (PLLA) emerges as a promising strategy due to its proven biocompatibility, adaptable biodegradability, and capacity to reproduce structural and biomechanical characteristics comparable to the host tissue’s extracellular matrix (ECM) [7]. However, despite its high crystallinity, chemical stability, and resistance to enzymatic degradation—which results in a prolonged reabsorption time—PLLA presents challenges. The hydrolytic degradation of PLLA generates acidic byproducts, such as lactic acid, which can acidify the in situ microenvironment, compromising the viability and differentiation of cells seeded on the scaffolds [8]. Additionally, the inherently hydrophobic nature of PLLA limits cell–material interactions and biological recognition at the material’s surface. This can lead to non-specific protein adsorption and undesirable reactions, thereby negatively impacting the biological response in vivo [9]. The typical degradation time for PLLA ranges from 30 to 40 weeks, depending on whether it is in an in vitro or in vivo environment [10].

To overcome the intrinsic limitations of PLLA and expand its spectrum of applications, its combination with other materials has been widely explored. In this context, graphene oxide (GO) stands out as a highly relevant carbonaceous material in contemporary research [11]. Its structure, rich in oxygen-containing functional groups (epoxy, hydroxyl, and carboxyl), confers upon GO remarkable hydrophilicity and a reversibly adjustable interlayer distance with increasing relative humidity, thereby facilitating its dispersion and functionalization [12].

The combination of PLLA and GO yields hybrid nanocomposites that synergistically enhance the performance of both materials [2]. These nanocomposites leverage the biodegradability and processability of PLLA to facilitate the fabrication of complex three-dimensional scaffolds, while simultaneously incorporating the enhancements conferred by GO in the composite’s mechanical properties, bioactivity, and, notably, its electrical conductivity [13]. This strategic combination confers a myriad of functional applications in tissue engineering and regenerative medicine, providing a favorable microenvironment for the morphofunctional restoration of various tissues, such as bone, cartilage, skin, and blood vessels [14].

In addition to scaffold development, a critical challenge in tissue engineering is the judicious selection of relevant cell types that adequately mimic the intrinsic characteristics of in vivo tissue, including their specific functional properties [15]. While tissue engineering research prioritizes the development of promising in vitro approaches for eventual translation to in vivo regeneration, understanding the natural response to tissue injury reveals a paradigm of spontaneous regeneration when the stromal tissue remains intact.

However, in cases of extensive injuries in non-regenerative tissues, where stromal tissue loss is significant, there is an urgent need to induce repair mechanisms, highlighting the importance of resident cells [16]. This study focuses on three cell lineages, whose relevance in the reconstruction of various organic tissues stems from their complementary and intrinsic biological roles within the stromal microenvironment and the repair process.

Human fibroblasts (FN1 cells)—cells of mesenchymal origin widely distributed in connective tissues—are crucial for tissue repair and remodeling. Their primary function lies in the synthesis and organization of the extracellular matrix (ECM), which provides the structural framework and the bioactive microenvironment necessary for angiogenesis and the subsequent deposition of new tissue [17]. In the stromal context, these fibroblasts act as essential components that shape the niche, responding to injury stimuli and contributing to the formation of granulation tissue—a precursor to repair—thus positioning them as key cells in stromal support and communication [18,19].

Mesenchymal stem cells (BMSCs), recognized as multipotent adult stem cells, exhibit the remarkable capacity for differentiation into various connective tissue cell subtypes. Such cells play a primary role in replenishing progenitor and tissue-specific cell populations, in addition to contributing significantly to the processes of angiogenesis, modulation of the inflammatory response, and consequently, tissue regeneration [20,21]. Within the stromal environment, MSCs act as multifunctional regulators, not only differentiating into tissue components but also exerting paracrine effects through the secretion of factors that modulate the activities of fibroblasts and endothelial cells. This promotes a pro-regenerative and anti-inflammatory environment critical for the restructuring of damaged stroma [22,23].

Human umbilical vein endothelial cells (HUVECs) are instrumental due to their proven ability to establish a functional vasculature, which is pivotal in facilitating vascularization within tissue engineering applications. This property is mediated by the secretion of angiogenic growth factors and intricate synergistic interaction with scaffold components [24]. Critically, HUVECs are fundamental components of the stromal vascular network, facilitating the transport of nutrients, oxygen, and trophic factors that are vital for the survival and proliferation of other stromal and parenchymal cells, in addition to modulating the initial inflammatory response. Consequently, the strategy of in vitro pre-vascularization, by seeding HUVECs into three-dimensional scaffolds, constitutes a promising approach to optimizing graft vascular integration and accelerating the formation of functional tissue [25,26].

Despite advancements in the quest for ideal scaffolds that mimic the intrinsic conditions of the extracellular matrix and optimize tissue synthesis, a gap in in-depth knowledge persists regarding the systematic interaction of novel biomaterials, such as the PLLA-GO nanocomposite, with different key cell populations during the early stages of tissue repair. Recognizing this gap, our research group proposes a rigorous methodology to evaluate the response of specific resident cell types to the new scaffold.

Thus, the main objective of this study was to investigate the cellular viability and proliferation, as well as the potential cytotoxicity, of the PLLA-GO nanocomposite using three distinct cell lines, namely human fibroblasts (FN1 cells), human umbilical vein endothelial cells (HUVECs), and mesenchymal stem cells (mBMSCs). Analyses were conducted with each cell type separately, avoiding co-cultures at this initial stage to ensure the reliability of the results for the proposed analyses and the unequivocal elucidation of specific material–cell interactions.

## 2. Materials and Methods

### 2.1. Cell Culture and Experimental Setup

Murine mesenchymal stem cells (mBMSCs) derived from C57BL/6J mice (Butantan Institute Animal Experimentation Ethics Committee—CEEA, Protocol n° 928/12), normal human fibroblasts (FN1 cells) (CEEA Protocol n° 921/06), and primary human umbilical vein endothelial cells (HUVECs) (ATCC CRL-1730, BSL 1^®^) utilized in this study belong to the cell bank of the Molecular Biology Laboratory at the Butantan Institute, under the supervision of Prof. Dr. Durvanei Augusto Maria.

Cell lines were cultured in RPMI-1640 medium (Cultilab, Campinas, São Paulo, Brazil), adjusted to pH 7.2 and supplemented with 10% heat-inactivated fetal bovine serum (FBS), 2 mM L-glutamine, and 1% antibiotic solution containing 10,000 IU/mL penicillin and 10 mg/mL streptomycin. Cells were cultured in 75 cm^2^ flasks under controlled humidity (5% CO_2_, 37 °C) until reaching >90% confluence.

Cell concentration and viability were assessed using a Neubauer chamber and a Trypan blue (1%) exclusion assay before experiments. This method enables the distinction between viable cells—characterized by plasma membrane integrity and consequent dye exclusion—and non-viable cells. Cell batches with ≥95% viability were selected for further assays.

To assess interactions with the PLLA-GO nanocomposite, the cells were suspended at a density of 1 × 10^4^ cells/mL. Exposure of the cells to the PLLA/GO nanocomposite was monitored for a period of up to 72 h, as outlined in the experimental protocol.

### 2.2. Preparation of PLLA-GO Nanocomposite

Graphene oxide (GO) was obtained via the chemical exfoliation of graphite (Nacional de Grafite Ltd.a^®^, São Paulo, Brazil), following the modified Hummers method [27]. The PLLA-GO nanocomposite (Mackgrape Laboratory, Mackenzie University, São Paulo, Brazil) was produced by incorporating a 0.2% (weight) fraction of GO into the poly(L-lactic acid) (PLLA; Evonik RESOMER^®^ L 210S) matrix. This was achieved using a Thermo Fisher Scientific Process 11 parallel twin-screw extruder (L/D 40, Waltham, MA, USA) under a controlled temperature profile with the following heating zones: Die (220 °C), Zone 8 (220 °C), Zone 7 (215 °C), Zone 6 (210 °C), Zone 5 (210 °C), Zone 4 (210 °C), Zone 3 (210 °C), and Zone 2 (200 °C). This process yielded filaments with a 1.75 mm diameter. The choice of a twin-screw extruder was particularly motivated by its high efficiency in dispersing nanofillers like GO within polymeric matrices, even at low concentrations. The intense shear forces and extensive mixing zones inherent to twin-screw extrusion promote a highly homogeneous distribution of GO within the PLLA matrix. This uniform dispersion is crucial for optimizing the final properties of the nanocomposite and ensures that GO does not excessively agglomerate, thereby preserving the desired characteristics essential for effective cellular interaction [28]. The selection of melt extrusion was primarily motivated by its inherent suitability for generating homogeneous filaments of precise dimensions, which serve as direct feedstock for Fused Deposition Modeling (FDM) 3D printing technologies. This method is recognized for its industrial relevance and scalability, consistently providing filaments with the necessary consistency and appropriate rheological properties for the additive manufacturing of intricate scaffolds [29]. Furthermore, melt extrusion presents significant advantages in terms of process efficiency and environmental impact. Compared to solvent-based methods like solution casting, melt extrusion eliminates the need for large volumes of organic solvents, thereby reducing associated costs, environmental risks, and critically, concerns regarding residual solvent in the final product, which is particularly beneficial for biomedical applications where material purity is paramount [30]. In addition, this method streamlines the fabrication process by allowing for the direct incorporation of GO (previously obtained via the Hummers method) into the PLLA matrix in a single mixing step, thereby simplifying the overall nanocomposite manufacturing process [31]. As demonstrated by characterization analyses, using the PLLA-GO nanocomposite as a scaffold results in a crystallinity degree of 42.5% [32].

The polymeric nanocomposite filaments were utilized as raw material for the production via 3D printing (Figure S1A) of discoidal scaffolds measuring 4 mm in diameter and 2 mm in height. These dimensions are compatible with the wells of a 96-well plate (U-bottom microplate, lot 1712201-1, Greiner Bio-One^®^ Ltd.a, Frickenhausen, Germany) (Figure S1B). The nanocomposite was sterilized with ethylene oxide during the manufacturing process. Additionally, prior to the experiments, the scaffolds were re-sterilized by autoclaving (121 °C, 15 psi, 20 min). To ensure the absence of contamination, a negative control was prepared by seeding PLLA-GO scaffolds without cells under the same experimental culture conditions (Figure S1C). These scaffolds were designed to enhance cellular adhesion and provide structural support for mBMSC, FN1 cell, and HUVEC cultures.

### 2.3. Cellular Characterization Techniques

This study evaluated the effects of the PLLA-GO nanocomposite on different cell types through in vitro experiments involving three distinct cell lines (see Section 2.1), namely normal human fibroblasts (FN1 cells), murine mesenchymal stem cells (mBMSCs), and human umbilical vein endothelial cells (HUVECs). Within each plate, cells from each lineage were divided into the following two experimental groups: a control group (48 wells), in which cells were cultured under standard conditions without PLLA-GO exposure, and an experimental group (48 wells), in which cells were exposed to discoidal PLLA-GO nanocomposite scaffolds measuring 4 mm in diameter and 2 mm in height.

#### 2.3.1. Evaluation of Cell Adhesion and Morphology via Inverted Optical Microscopy

Cell adhesion and morphology on PLLA-GO substrates were assessed by inverted microscopy, using an LSM 780-NLO confocal microscope (Zeiss, Oberkochen, Germany) coupled to an Axio Observer Z.1 inverted system (Carl Zeiss AG, Jena, Germany), equipped with a 40× objective (Carl Zeiss AG, Jena, Germany) (scale bar: 100 µm). Cell cultures were maintained under standard incubation conditions (37 °C, 5% CO_2_ in a humidified atmosphere) throughout image acquisition. Images were acquired at predefined time intervals (24, 48, and 72 h) using an Axiocam 506 color digital camera (Carl Zeiss AG, Germany) and ZEN 2 (Blue edition) software (Carl Zeiss AG, Jena, Germany).

#### 2.3.2. Morphological Characterization by Scanning Electron Microscopy (SEM)

The surface morphology of cells in the presence of PLLA-GO scaffolds was investigated by scanning electron microscopy (LEO 435VP, Carl Zeiss Microscopy, Oberkochen, Germany) after 72 h. Fragments containing the PLLA-GO nanocomposite and the various cells were prepared by primary fixation in 3% (*v*/*v*) glutaraldehyde in 0.1 M sodium phosphate buffer (PBS), followed by post-fixation in 1% osmium tetroxide (OsO_4_). Sequential dehydration was performed using a gradual series of ethanol (80%, 90%, and 100%, with two 5 min immersions each), culminating in critical point drying with liquid carbon dioxide (CO_2_). The LEICA EM CPD300 (Leica Microsystems GmbH, Wetzlar, Germany) was used to preserve cellular ultrastructure.

Dried samples were mounted on aluminum stubs with conductive carbon tape and sputter-coated with a thin gold layer using an EMITECH^®^ K550 metallizer (Quorum Technologies Ltd., Ashford, UK), operated at 35 mA for 120 s. The conductive layer thickness was optimized to enhance secondary electron emission and improve the signal-to-noise ratio while preserving spatial resolution (Figure S2A–C).

Images were acquired using a Leo 435VP scanning electron microscope (Leo, Cambridge, UK) operated in secondary electron mode, with acceleration voltages and beam currents optimized for each experimental condition (Figure S2D). Image processing was conducted at the Advanced Center for Image and Molecular Diagnostics (CADIM), Faculty of Veterinary Medicine and Animal Science, USP. The magnifications used for documentation were specified in the corresponding figures (Section 3).

#### 2.3.3. Flow Cytometric Analysis of Cell Cycle Distribution

The distribution of cell cycle phases was determined by flow cytometry through the quantification of cellular DNA content. Cells adhered to the scaffolds were trypsinized and centrifuged at 1500 rpm for 5 min. The resulting cell pellet was then resuspended in a solution containing 70% (*v*/*v*) alcohol and RNase alcohol and stored at −20 °C for 24 h for fixation. Before analysis, fixed samples were centrifuged at 1500 rpm for 5 min, and the pellet was resuspended in 200 µL of FACS buffer (Fluorescence-Activated Cell Sorting; typically PBS with 1–2% BSA and EDTA). The cells were then permeabilized by adding 20 µL of Triton X-100 (Sigma-Aldrich, St. Louis, MO, USA), and the cellular DNA was stained with 50 µg/mL of propidium iodide (PI) (Sigma-Aldrich, St. Louis, MO, USA). Samples were incubated at room temperature for 30 min, protected from light. Following incubation, samples were transferred to flow cytometry tubes and analyzed using a FACSCalibur flow cytometer (BD Biosciences, San Jose, CA, USA) in the FL2-H fluorescence channel, set to acquire 10,000 events per sample.

The results were analyzed using ModFit LT software version 3.2 (Verity Software House, Topsham, ME, USA). Histograms display the percentage of cells distributed across distinct cell cycle phases—Sub-G1, G0/G1, S, and G2/M—expressed as mean ± standard deviation (SD).

#### 2.3.4. Cell Proliferation Analysis by Generation Tracking with CFSE

The proliferation rate of FN1 cells, mBMSCs, and HUVECs was evaluated using the fluorescent dye carboxyfluorescein succinimidyl ester (CFSE-DA Thermo Fisher, KITC34571, Waltham, MA, USA). In 24-well plates, 1 × 10^4^ cells/mL were seeded in complete culture medium. Then, 2 µL of diluted CFSE-DA was added directly to each well containing 500 µL of culture medium, for both the control group (without PLLA-GO) and the PLLA-GO-exposed group (cells + nanocomposite), resulting in a final CFSE concentration of 19.92 µM.

The PLLA-GO-exposed and control groups were incubated for 24 h under controlled conditions (37 °C, 5% CO_2_) to facilitate CFSE incorporation. The labeling reaction was naturally terminated by washing and subsequent incubation. After the incorporation period, the supernatant was removed, and 250 µL of trypsin were added to each well to dissociate the adherent cells. After 5 min at 37 °C, trypsin was inactivated with double the volume of complete culture medium. Labeled cells were transferred to centrifuge tubes and spun at 1500 rpm for 5 min, after which the supernatant was discarded.

Next, the cells were resuspended in 200 µL of 4% paraformaldehyde for fixation and stored in the refrigerator until the day of reading. On the analysis day, the cells were re-centrifuged at 1500 rpm for 5 min, and the supernatant was removed. The cells were then resuspended in 200 µL of fluorescence-activated cell sorting (FACS) buffer.

The readings were performed on a FACScanto flow cytometer (BD) with the acquisition of 10,000 events per sample. CFSE fluorescence intensity histograms were analyzed using ModFit LT 5.0 software (Verity Software House, Topsham, ME, USA). This software was used to model cell population dynamics across successive division generations based on CFSE fluorescence intensity distribution. Through sophisticated curve-fitting algorithms applied to fluorescence peaks corresponding to each generation, it was possible to quantify the percentage of cells in each generation and estimate parameters such as the cell proliferation index.

#### 2.3.5. Analysis of Mitochondrial Electrical Potential by Flow Cytometry

The mitochondrial membrane potential (ΔΨm) was assessed using the lipophilic cationic dye Mito-Red (TMRE—tetramethylrhodamine ethyl ester; Sigma-Aldrich, St. Louis, MO, USA), a fluorochrome specific for labeling active mitochondria. After the respective treatment periods, samples of FN1 cells, mBMSCs, and HUVEC cells were processed for the analysis of mitochondrial electrical potential.

The cells were centrifuged at 1500 rpm for 5 min, and the supernatant was removed. The cell pellet was then resuspended in 100 µL of RPMI-1640 culture medium containing 200 nM of Mito-red dye (Sigma-Aldrich, St. Louis, MO, USA). Samples were incubated at 37 °C and 5% CO_2_ for 1 h to facilitate dye incorporation. Following incubation, cells were centrifuged again at 1500 rpm for 5 min, after which the supernatant was discarded, and the pellet resuspended in 100 µL of FACS Flow buffer (PBS with 1–2% BSA and EDTA). The reading and analysis of Mito-red staining in the cells were performed on a FACSCanto^®^ II flow cytometer (Becton, Dickinson and Company, BD Biosciences, Franklin Lakes, NJ, USA), using the 488 nm laser for excitation and emission detection at FL1-H fluorescence intensity (10,000 events). Histograms were generated and analyzed using CellQuest^®^ software (version 2.0, Becton, Dickinson and Company, BD Biosciences, San Jose, CA, USA). Mitochondrial membrane potential (ΔΨm) was quantified by measuring the fluorescence intensity of the dye, where higher intensity correlated with increased mitochondrial activity.

#### 2.3.6. Analysis of Inflammatory Cytokines by Flow Cytometry (CBA)

Cytokine quantification was performed using the BD Cytometric Bead Array (CBA) system (BD Biosciences, San Jose, CA, USA), with specific kits applied according to the cellular model analyzed. For the human cell lines—FN1 cells (fibroblasts) and HUVECs (endothelial cells)—the murine cell line, mBMSCs (murine mesenchymal stem cells), the following cytokines were measured: IL-12p70 (Interleukin-12p70), TNF-α (Tumor Necrosis Factor alpha), IL-10 (Interleukin-10), IL-6 (Interleukin-6), IL-1β (Interleukin-1 beta), IL-8 (Interleukin-8), IFN-γ (Interferon Gamma), and MCP-1 (Monocyte Chemoattractant Protein-1). The specific kits utilized for each cell line are detailed in Table 1.

FN1 cells, HUVECs, and mBMSCs were plated in 6-well plates at a density of 2 × 10^5^ cells/well. After a 24 h incubation, culture supernatants were exposed to the PLLA-GO nanocomposite for 72 h before being harvested and stored at −80 °C until analysis. The CBA assay was performed according to the manufacturer’s instructions, with minor modifications. Specific capture microbeads for each cytokine were mixed by combining 10 µL of each microbead suspension to prepare the multiplex bead mix. Then, 50 µL of the mixed microbead solution, 50 µL of the cell culture supernatant (or cytokine standard), and 50 µL of the PE Detection Reagent (phycoerythrin-conjugated anti-cytokine antibodies, specific for the cell species) were added to the respective assay tubes. The tubes were incubated at room temperature for 2 h, protected from light, followed by the addition of 1 mL of Wash Buffer and centrifugation at 1500 rpm for 5 min. The supernatant was carefully aspirated and discarded, and the microbead pellet was resuspended in 300 µL of FACSFlow solution. Samples were acquired on a FACSCanto II flow cytometer (Becton, Dickinson and Company, BD Biosciences, USA), and data were analyzed using BD FCAP Array software (BD Biosciences, version 3.0.1), which incorporates dedicated CBA analysis modules.

### 2.4. Statistical Analysis

Statistical analyses of data obtained from the FN1 cell, mBMSCs, and HUVECs exposed to the PLLA-GO nanocomposite were conducted using GraphPad Prism 8, adopting a significance threshold of α = 0.05. Data normality was assessed via the Shapiro–Wilk test. Comparisons of cell cycle phase distributions between control and treated groups were performed using an unpaired Student’s *t*-test. The effects of PLLA-GO treatment on generation-specific cell counts, proliferative index, and mitochondrial membrane potential were analyzed using paired Student’s *t*-tests. Cytokine concentrations were likewise assessed via paired *t*-tests.

## 3. Results

### 3.1. Assessment of Cell Adhesion and Morphology by Inverted Optical Microscopy

**Figure 1:**
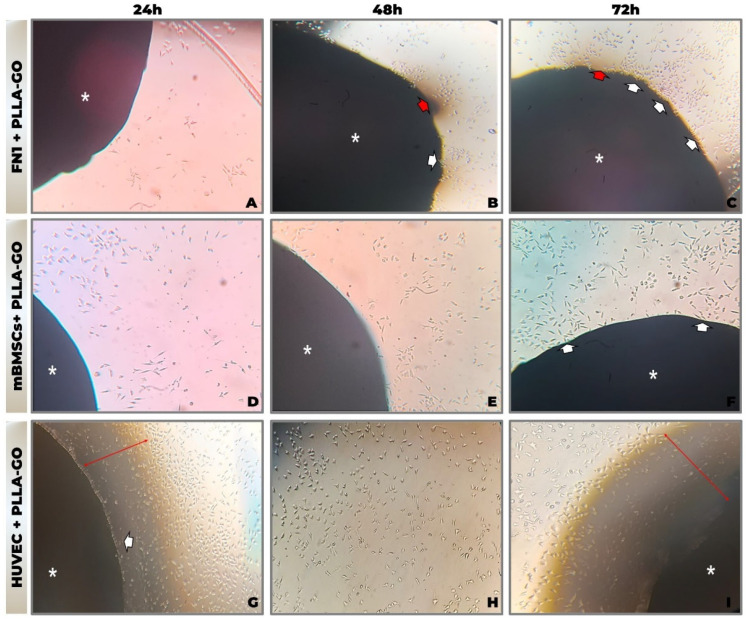
Morphological analysis by inverted optical microscopy of cell line interaction with PLLA-GO nanocomposite. (**A**,**D**,**G**) 24 h; (**B**,**E**,**H**) 48 h; (**C**,**F**,**I**) 72 h. Photomicrographs of FN1 cells (**A**–**C**), mBMSCs (**D**–**F**), and HUVECs (**G**–**I**) cultured in the presence of PLLA-GO. Legend: Asterisk (*): Indicates the PLLA-GO nanocomposite. White arrows: Point to cells at the interface or migrating towards the nanocomposite. Red arrows: Indicate areas of contact or cellular concentration at the nanocomposite interface. Objective: 10× (magnification: ×100).

Sequential morphological analysis by inverted optical microscopy (at 24, 48, and 72 h) revealed efficient adhesion and progressive proliferation of FN1 cells, mBMSCs, and HUVECs cultured in contact with PLLA-GO nanocomposites, without morphological evidence of cytotoxicity (Figure 1A–I). Temporal analysis demonstrated cell-type-specific interaction patterns with the biomaterial surface.

In the FN1 cell cultures, elongated, spindle-shaped fibroblasts adhered and accumulated near the PLLA-GO interface (Figure 1C). Cellular projections extended toward the scaffold, and minor variations in fibroblast morphology were observed in the surrounding area, suggesting possible material fragmentation.

mBMSCs initially presented a rounded morphology, progressively elongating and orienting toward the scaffold. Cell density increased over time, culminating in direct adhesion to the PLLA-GO surface (Figure 1D–F).

HUVECs maintained a cobblestone-like morphology, forming a confluent monolayer around the nanocomposite. Cells extended fine projections toward the PLLA-GO, with increased density noted over time in the contact zone (Figure 1G–I).

Across all cell lines, no morphological indicators of cytotoxicity—such as membrane blebbing, excessive detachment, or cellular disintegration—were observed. Furthermore, the absence of aggregation or microbial artifacts underscores the sterility and stability of the culture system, reinforcing the apparent primary biocompatibility of the PLLA-GO nanocomposite.

### 3.2. Cellular Morphological Characterization by Scanning Electron Microscopy (SEM)

**Figure 2:**
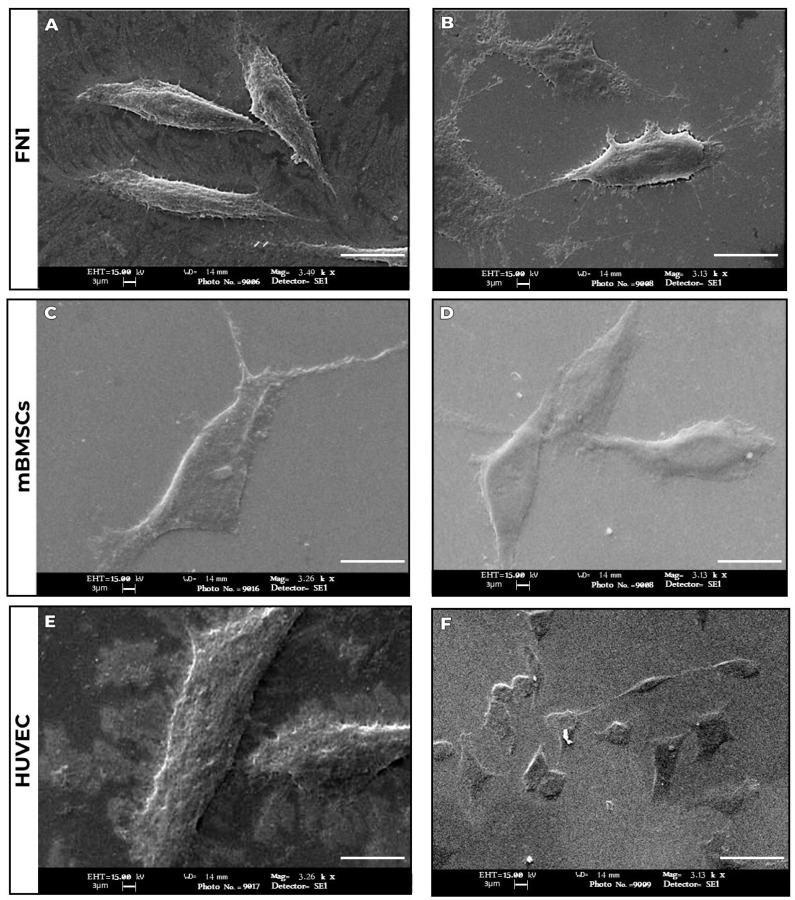
Cell morphology of cell lines exposed to PLLA-GO nanocomposite after 72 h of culture, analyzed by scanning electron microscopy (SEM). FN1 cells (**A**,**B**) exhibit a spindle-shaped morphology with filopodial extensions. mBMSCs (**C**,**D**) exhibit an irregular polygonal shape with lamellipodial extensions. HUVECs (**E**,**F**) demonstrate a pavement-like morphology and confluent organization. Progressive cell spreading is observed in all cultures. SEM image acquisition parameters: Secondary electron detector (SE1); acceleration voltage of 15.00 kV; scale bar = 3 µm. Magnifications were 3.49 K X for images (**A**–**E**), 3.13 K X for (**B**–**F**), 3.26 K X for (**C**), and 3.13 K X for (**D**).

Scanning electron microscopy (SEM) analysis demonstrated that the PLLA-GO nanocomposite influenced cellular organization in a lineage-dependent manner (Figure 2A–F).

The nanocomposite exhibited a relatively uniform surface with no significant graphene oxide (GO) agglomeration, reflecting the effectiveness of the optimized melt extrusion process [33]—a crucial feature for favorable cellular interactions.

In the FN1 cell cultures (Figure 2A,B), cells displayed an elongated morphology with fine cytoplasmic extensions and irregular surfaces, consistent with active extracellular matrix synthesis. mBMSCs (Figure 2C,D) exhibited notable clustering behavior and pronounced intercellular junctions, indicative of regenerative signaling. HUVECs (Figure 2E,F) retained their characteristic cobblestone morphology, showing increased contact and membrane projections.

Across all groups, no morphological signs of cytotoxicity—such as cellular shrinkage, fragmentation, or dedifferentiation were observed. The distinct interaction patterns observed for each cell type suggest that PLLA-GO not only supports cell viability but also modulates the local microenvironment to favor lineage-specific biological activity.

### 3.3. Flow Cytometric Analysis of Cell Cycle Distribution Cell Cycle Distribution Analysis

Table 2 presents a detailed comparative analysis of cell cycle phases (G0/G1, S, G2/M) and the debris fraction for the FN1 cells, mBMSCs, and HUVECs in both the control and PLLA-GO-treated groups. Standard deviations (SDs) and *p*-values indicate the statistical significance of observed differences.

**Figure 3:**
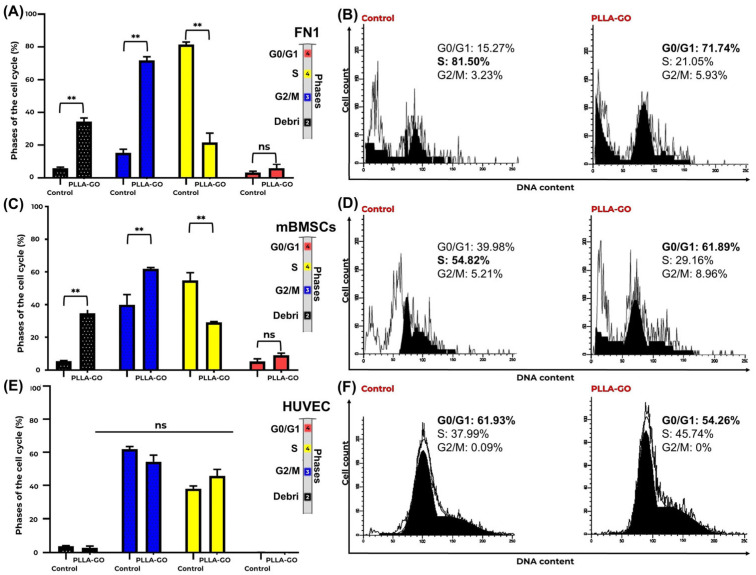
Cell cycle analysis of FN1, mBMSCs, and HUVECs in response to PLLA-GO nanocomposite. Comparative bar graphs illustrate the percentage of FN1 cells (**A**), mBMSCs (**C**), and HUVECs (**E**) in the G0/G1, S, and G2/M phases for both control and PLLA-GO groups. For FN1 cells and mBMSCs, the presence of the nanocomposite induced a significant increase in the proportion of cells in the G0/G1 phase and a decrease in the S phase (*p* ≤ 0.05). In contrast, HUVECs showed no significant alterations in cell cycle phases (*p* ≥ 0.05). Error bars represent the standard deviation. Asterisks (**) indicate statistically significant differences (*p* ≤ 0.05). Representative histograms of DNA content for FN1 cells (**B**), mBMSCs (**D**), and HUVECs (**F**) from both control and PLLA-GO groups are also presented. The peaks in these histograms correspond to the different cell cycle phases. In the control groups of FN1 and mBMSCs, the main peak is located in the S phase region. Conversely, in the PLLA-GO group, the main peak shifts to the G0/G1 phase region. For HUVECs, the main peak remains in the G0/G1 region in both groups.

mBMSCs also demonstrated significant redistribution across the cell cycle phases following PLLA-GO exposure. A significant increase in G0/G1 phase cells (*p* = 0.039) was observed, coupled with a significant reduction in the S phase (*p* = 0.027), indicating a potential modulation in cell cycle progression and a decrease in DNA synthesis activity. Similar to the FN1 cells, a significant increase in the debris fraction was noted in the PLLA-GO-exposed mBMSCs (*p* = 0.005) (Table 2, Figure 3C,D).

In contrast to the FN1 cells and mBMSCs, HUVECs showed no significant alteration in their cell cycle distribution following PLLA-GO exposure. Both groups maintained their primary distribution across the G0/G1 and S phases, with minimal presence in G2/M, and no significant differences were found between control and treated groups for any cell cycle phase (*p* ≥ 0.05) or in the proportion of debris (Table 2, Figure 3E,F).

### 3.4. Cell Proliferation Analysis by Generation Tracking with CFSE Cell Proliferation Profiles

CFSE proliferation analysis revealed distinct division patterns across the cell lines (Table 3). For both the FN1 cells and mBMSCs, clear progression through generations 2, 3, and 4 was observed. HUVECs exhibited divisions spanning from the second to the tenth generation, with an apparent absence of the 7th generation, followed by the detection of populations in later generations by the experiment’s end.

**Figure 4:**
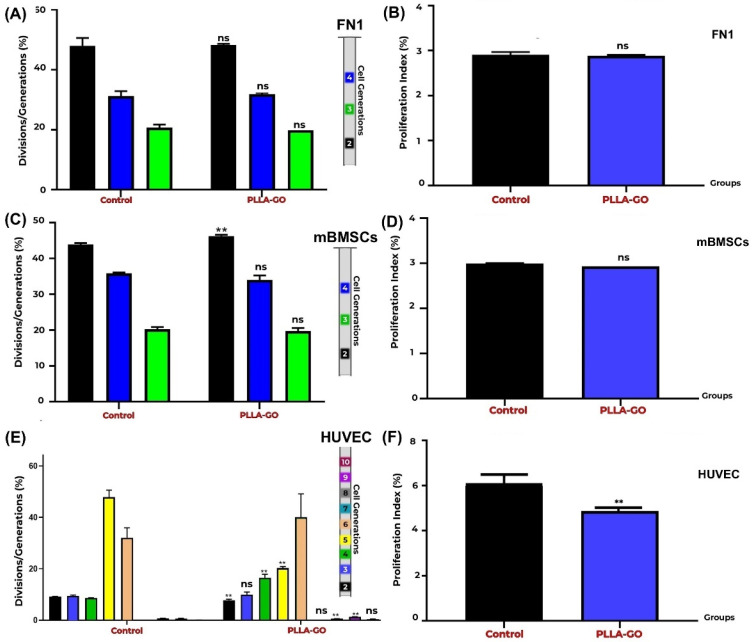
Proliferation analysis of FN1 cells, mBMSCs, and HUVECs by CFSE. Generation Tracking Comparative bar graphs show the percentage of FN1 cells (**A**), mBMSCs (**C**), and HUVECs (**E**) across different generations (FN1: 2nd–4th; mBMSCs: 2nd–4th; HUVECs: 2nd–10th) in both control and PLLA-GO groups. For FN1 cells, there was no significant difference between the groups in any of the analyzed generations (*p* ≥ 0.05) (**A**). In mBMSCs, a significant increase in the percentage of cells in the 2nd generation was observed in the PLLA-GO group (*p* ≤ 0.05), with no significant differences in the other generations (**B**). For HUVECs, the PLLA-GO group showed significant differences in the percentage of cells across various generations, including a decrease in initial generations and an accumulation in more advanced generations, with the absence of the 7th generation (*p* ≤ 0.05) (**E**). The cellular proliferation index for the FN1 cells (**B**), mBMSCs (**D**), and HUVECs (**E**) in the control and PLLA-GO groups was also assessed. In panels B, D, and F, black bars represent the Control group and blue bars represent the PLLA-GO group. There was no significant difference in the proliferation index for the FN1 cells and mBMSCs between the groups (*p* ≥ 0.05) (**B**,**D**). However, HUVECs exposed to PLLA-GO presented a significant decrease in the cellular proliferation index (*p* ≤ 0.05) (**F**). Error bars represent the standard deviation. “ns” indicates no statistically significant difference (*p* ≥ 0.05), and asterisks (**) indicate statistically significant differences (*p* ≤ 0.05).

The FN1 cells exposed to PLLA-GO maintained a replicative dynamic equivalent to that of the control group, with no statistically significant difference (*p* ≥ 0.05) in their generational distribution (Table 3; Figure 4A,B). This indicates a similar proliferative progression pattern between the groups, suggesting that PLLA-GO treatment did not impact the ability of the FN1 cells to divide and progress through the analyzed cell generations.

Analysis of mBMSC generational distribution after PLLA-GO exposure revealed a significant effect on initial proliferative progression (*p* = 0.003). However, this initial difference was not sustained in subsequent generations, as the distribution of cells in generations 3 and 4 was similar between the control and treated groups. The absence of significant differences in later generations suggests that while PLLA-GO may influence the kinetics of the first cell division, it does not affect the overall long-term proliferative capacity of mBMSCs. This pattern implies a transient effect of PLLA-GO on the cell cycle, possibly related to early regulatory changes that are not sustained across multiple cell divisions (Table 3; Figure 4C,D).

For HUVECs, proliferation analysis was possible from the second to the tenth generation, noting that data for the seventh generation were not quantifiable due to very low population representation (Table 3). PLLA-GO was found to significantly alter the generational distribution of HUVECs. Progression to the second generation was accelerated, demonstrated by a significant reduction in cells at this generation (*p* = 0.0005). Conversely, PLLA-GO also caused a significant accumulation of cells in generations 4 (*p* = 0.005) and 10 (*p* = 0.03). This complex, multiphasic pattern suggests that PLLA-GO influences HUVEC cell proliferative kinetics, with an initial acceleration followed by an accumulation in more advanced generations (Table 3; Figure 4E,F).

### 3.5. Analysis of Mitochondrial Electrical Potential by Flow Cytometry Effects on Mitochondrial Electrical Potential

To evaluate the impact of PLLA-GO treatment on the mitochondrial electrical potential (ΔΨm)) of the FN1 cells, mBMSCs, and HUVECs, the proportion of cells with active and inactive mitochondrial potential was quantified by flow cytometry. Table 4 summarizes the results across all cell lines under different conditions.

**Figure 5:**
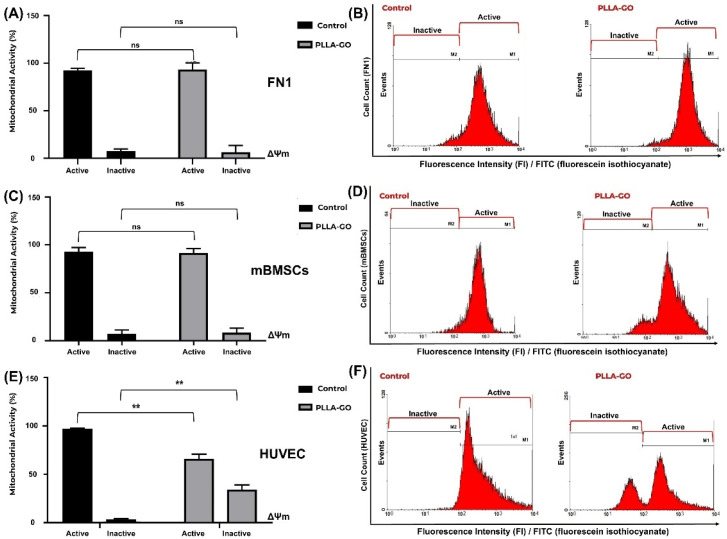
Analysis of mitochondrial electrochemical potential (ΔΨm) in FN1 cells, mBMSCs, and HUVECs in response to PLLA-GO nanocomposite. Comparative bar graphs illustrate the percentage of cells with active and inactive mitochondrial potential in the control and PLLA-GO groups for the FN1 cells (**A**), mBMSCs (**C**), and HUVECs (**E**). FN1 cells and mBMSCs predominantly maintained an active ΔΨm in both control and PLLA-GO groups, with no statistically significant differences (*p* ≥ 0.05). In contrast, HUVECs exposed to PLLA-GO showed a significant reduction in the percentage of cells with active mitochondrial potential and a substantial increase in cells with inactive potential (*p* ≤ 0.05). Representative histograms of FITC dye fluorescence intensity (FI) indicate the distribution of cells with active and inactive mitochondrial potential in control and PLLA-GO groups for the FN1 cells (**B**), mBMSCs (**D**), and HUVECs (**F**). Region M1 represents cells with active mitochondrial potential (high fluorescence, indicative of polarized mitochondria), while region M2 corresponds to cells with inactive mitochondrial potential (low fluorescence, reduced ΔΨm). The histograms demonstrate the maintenance of active potential for FN1 cells and mBMSCs, and a clear transition to the inactive state in HUVECs with PLLA-GO. “ns” indicates no statistically significant difference (*p* ≥ 0.05); asterisks (**) indicate statistically significant differences (*p* ≤ 0.05).

Analysis of ΔΨm in FN1 cells revealed that the majority of cells in both control and PLLA-GO exposed groups maintained active mitochondrial potential (Table 4, Figure 5A,B). The results indicate no significant alteration in the FN1 cells’ mitochondrial electrical potential after PLLA-GO exposure, suggesting that the treatment did not induce significant mitochondrial dysfunction.

Similarly, mBMSCs exposed to PLLA-GO maintained a ΔΨm equivalent to that of the control, with no significant difference in the proportion of cells with reduced ΔΨm (Table 4, Figure 5C,D). Consistent with the FN1 cell observations, a slight, non-significant widening of the histogram’s left tail in the PLLA-GO group suggests transient mitochondrial depolarization compatible with physiological fluctuations. These findings collectively corroborate that the nanocomposite does not compromise the energetic integrity or viability of FN1 cells and mBMSCs.

In contrast to the previous cell lines, the exposure of HUVEC cells to PLLA-GO induced a significant reduction in mitochondrial electrical potential (ΔΨm) (*p* ≤ 0.05) (Table 4, Figure 5E,F). The percentage of cells with active potential decreased by 32.19%, while the proportion of cells with inactive mitochondria substantially increased by approximately 1026.32% (*p* ≤ 0.05). These results strongly suggest that PLLA-GO treatment can lead to significant mitochondrial dysfunction in HUVEC cells, with direct implications for their function and viability.

### 3.6. Analysis of Inflammatory Cytokines by Flow Cytometry (CBA)

Analysis of inflammatory cytokines via flow cytometry (CBA) revealed distinct response profiles to PLLA-GO treatment across the investigated cell lines (FN1 cells, mBMSCs, and HUVEC) when compared to their respective controls (Table 5).

**Figure 6:**
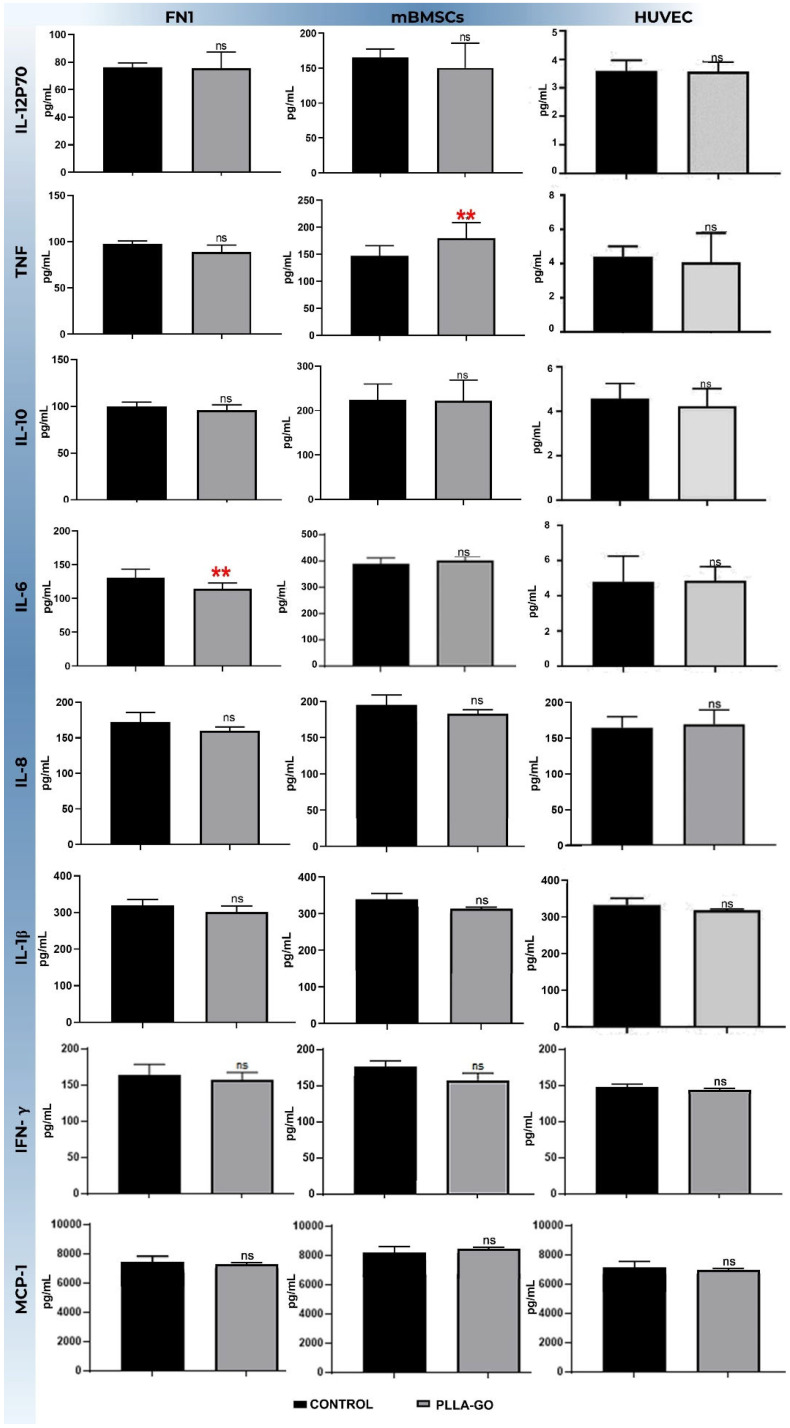
Analysis of inflammatory cytokine levels in FN1 cells, MBMSCs, and HUVECs after PLLA-GO exposure the bars represent the mean concentration (±standard deviation) of different cytokines (IL-12p70, IL-10, IL-8, 1L-1β, IFN-γ and MCP-1) in picograms per milliliter (pg/mL) for the control group (black bars) and the PLLA-GO-exposed group (gray bars) in each cell line. Statistical significance was determined by appropriate tests, and significant differences are indicated by asterisks: ** (*p* ≤ 0.05). “ns” indicates a non-significant difference (*p* ≥ 0.05).

In the FN1 cells, a significant reduction was observed in the IL-6 levels (*p* = 0.019). For TNF-α, an increasing trend was noted, but the difference did not reach statistical significance (*p* = 0.052). Other analyzed cytokines, including IL-12p70, IL-10, IL-8, IL-1β, IFN-γ, and MCP, showed no significant changes between the PLLA-GO-treated group and the control (Table 5; Figure 6).

In contrast, mBMSCs exposed to PLLA-GO showed a significant increase in TNF-α production (*p* = 0.03). All other cytokines analyzed in this cell line—IL-12p70, IL-6, IL-10, IL-8, IL-1β, IFN-γ, and MCP-1—did not show statistically significant differences compared to the control group (Table 5; Figure 6).

For the HUVECs, exposure to PLLA-GO resulted in a significant reduction in both TNF-α (*p* = 0.05) and IL-6 levels (*p* = 0.02). The other cytokines, including IL-12p70, IL-10, IL-8, IL-1β, IFN-γ, and MCP-1, showed no significant changes in their levels after PLLA-GO exposure compared to the control (Table 5; Figure 6).

## 4. Discussion

Despite significant advancements in designing nanocomposites for tissue engineering, a persistent knowledge gap remains in fully understanding the systematic response of different key cell populations—such as human fibroblasts (FN1 cells), human umbilical vein endothelial cells (HUVECs), and mesenchymal stem cells (mBMSCs)—to novel biomaterials like the PLLA-GO nanocomposite during tissue repair stages. Comprehending this response is crucial for the rational design of biomaterials with specific and optimized applications in the context of tissue repair.

This study addresses this gap by systematically investigating the viability, proliferation, cytotoxicity, morphological organization, and inflammatory response profile of these cell lines following exposure to PLLA-GO. The data presented here provide a multifaceted profile of the PLLA-GO–cell interaction, revealing distinct, cell-specific behaviors critical for evaluating the biocompatibility and potential application of this biomaterial in tissue engineering strategies.

In line with the objective of evaluating material–cell interaction, morphological analyses by both inverted optical microscopy and scanning electron microscopy (SEM) generally revealed primary biocompatibility of the PLLA-GO nanocomposite with all three cell lines studied. This was characterized by efficient adhesion and progressive proliferation, with typical preservation of the exposed cell lines’ morphologies [32,34,35].

The incorporation of graphene oxide (GO) into polymers like poly(L-lactide) (PLLA) is well documented, solidifying its importance in advanced biomaterial development. Evidence indicates that incorporating 0.4 wt% GO can increase mechanical strength by up to 44%, while concentrations of 1.2% can yield electrical conductivity above 1 mS/cm [36]. Additionally, its biocompatibility has been extensively validated, with studies reporting cell viability exceeding 70% [37], further strengthening its potential for applications in tissue engineering [38,39]. However, despite the expected synergistic characteristics, the specific 3D printing fabrication process and the construction of the nanocomposite are critical factors that can determine changes in properties that are crucial for cell interaction. Variations in surface topography, pore interconnectivity, hydrophilicity, and even residual toxicity can be induced by processing conditions. These alterations directly impact cell adhesion and proliferation, influencing anchoring, migration, and nutrient access [40,41]. Consequently, these peculiarities inherent to the nanocomposite’s fabrication step can infer changes in the observed results in our study, manifesting distinctly across different cell lines, even within a context of primary biocompatibility. Importantly, the long-term stability and potential toxicity of graphene-based materials remain active areas of investigation.

The chosen method for nanocomposite fabrication—melt extrusion—represents a highly optimized and compatible approach for the intended biological applications, particularly when synergistically combined with 3D printing technologies. Specifically, the capability of melt extrusion to produce high-quality filaments for Fused Deposition Modeling (FDM) inherently optimizes the subsequent scaffold fabrication. This is crucial as 3D printing enables the creation of complex geometries and customized scaffolds with precisely controlled porous architectures, which are essential prerequisites for successful tissue engineering and regenerative medicine strategies [42]. The inherent versatility and precision offered by 3D printing, combined with the robust material properties achieved through melt extrusion, establish a strong foundation for developing advanced biomaterials.

A key advantage of melt extrusion for biomedical applications lies in its ability to avoid the massive use of organic solvents, which significantly simplifies the biocompatibility assurance process. While high processing temperatures (200–220 °C) during extrusion necessitate careful control to prevent potential degradation of PLLA or reduction in GO, the optimized residence time and temperature profile conditions within the extruder are meticulously managed to minimize such adverse effects, thereby preserving the molecular and functional integrity of the components. Furthermore, the post-production sterilization procedures, specifically ethylene oxide sterilization and autoclaving (as detailed in the Section 2), further complement the overall biological safety of the material [43].

Moreover, the homogeneous dispersion of GO achieved through the melt extrusion process is essential for ensuring that the bioactive properties of GO are presented uniformly to the cells. This uniform presentation prevents the formation of aggregates that could otherwise induce heterogeneous cellular responses or localized cytotoxicity, thereby promoting consistent and predictable cell–material interactions [44].

Beyond its intrinsic compatibility with biological applications, melt extrusion offers distinct advantages over alternative synthesis techniques, such as solution casting, significantly impacting the final physicochemical properties of the material. To provide comprehensive evidence supporting the chemical characterization of our nanocomposite, we have specifically considered the implications of melt extrusion in terms of mechanical strength, swelling behavior, morphological features, and thermal stability, drawing comparisons with established literature. When optimized for nanofiller dispersion, melt extrusion can effectively enhance the mechanical properties of PLLA, such as Young’s modulus and tensile strength, especially with GO incorporation, as shear forces promote better GO lamellae orientation and interfacial interaction, resulting in significant reinforcement corroborated by the extensive literature for PLLA/GO nanocomposites suitable for structural scaffolds; the robustness of our 3D printing filaments serves as a practical indication of this mechanical integrity [45]. Furthermore, melt extrusion typically yields dense materials with low intrinsic porosity (before 3D printing), influencing the PLLA-GO nanocomposite’s swelling behavior, which is primarily controlled by GO’s hydrophilicity and PLLA’s crystallinity (42.5%). Extruded materials generally exhibit lower swelling compared to porous films made by solution casting, which is advantageous for dimensional stability in biological environments, a fact corroborated by the observed stability of our scaffolds in cell culture medium during viability and proliferation assays [46]. Regarding thermal stability, while high extrusion temperatures (200–220 °C) are a critical consideration, PLLA has an adequate processing window, and GO can even act as a nucleating agent, enhancing PLLA’s thermal stability. The literature confirms that PLLA/GO nanocomposites can be extruded without significant property loss under carefully controlled conditions, and the stability of our nanocomposite during 72 h cell viability assays at 37 °C provides practical validation of its integrity under physiological conditions [28].

FN1 cells exhibited adhesion and spreading behavior characteristic of fibroblasts on biocompatible substrates [47], forming intercellular networks and maintaining their typical morphoarchitecture in the presence of PLLA-GO—comparable to that of the control group. This in vitro behavior reflects the high phenotypic plasticity inherent to fibroblasts, widely documented as a mechanism of functional adaptation to microenvironmental variation and exogenous cues [48]. Nonetheless, a mild degree of cell dispersion was observed in some FN1 cultures after 72 h, raising the possibility of a subtle material-induced stress response, despite the absence of morphological signs of acute cytotoxicity [49].

Likewise, mBMSCs in contact with PLLA-GO retained morphological integrity, with no significant deviations from control cultures. Local variations in cell density may reflect the topographical influences of the biomaterial’s nanoscale architecture. The observed self-organization and increased intercellular junctions are consistent with morphogenetic signaling events associated with regeneration, wherein spatial cell proximity and communication orchestrate niche formation and initiation of differentiation programs [28]. The preservation of morphology and strengthening of homotypic interactions further support the cytocompatibility of PLLA-GO for mBMSCs, aligning with prior studies on PLLA-GO biointerfaces [30,32].

In HUVEC cultures, the typical cobblestone morphology and enhanced cell–cell interactions were clearly observed under both optical and SEM imaging. This suggests that PLLA-GO may support endothelial organization and positively modulate adhesion and intercellular communication [50], both essential for the formation of functional vascular networks [51].

Across all cell lines, the absence of adverse morphological changes—such as apoptotic bodies or dedifferentiation—and the maintenance of lineage-specific features underscore the biocompatibility of PLLA-GO and its ability to favorably modulate the cellular microenvironment [52]. The minor morphological change noted in FN1 cultures, possibly associated with material fragmentation, raises the hypothesis that although the scaffold is biocompatible, it may be subject to degradation. If excessive or uncontrolled, such degradation could influence long-term cellular behavior [53].

Complementary scanning electron microscopy (SEM) analysis revealed the nanocomposite’s surface topography and cell distribution across all three cell lines. SEM highlighted the formation of membranous extensions and cellular clusters on both the scaffold surface and within its internal pores. Given that PLLA is known to form porous scaffolds [32], it is hypothesized that the structural architecture of the composite is dictated by the intrinsic porosity of PLLA. Such pores likely play a key role in facilitating cell infiltration, intercellular connectivity, and spatial occupancy within the scaffold [54].

These findings are consistent with previous studies indicating that oxygen-containing functional groups on the GO surface can interact non-specifically with cell membrane molecules through weak chemical interactions, such as electrostatic, hydrogen, or ionic bonds [55]. The incorporation of GO into the PLLA matrix enhances the nanocomposite’s specific surface area, thereby increasing available binding sites [55,56]. These interactions are known to activate intracellular signaling cascades—including the MAPK, PI3K/AKT, JAK/STAT, and NF-κB pathways—ultimately promoting cellular adhesion, proliferation, and differentiation [56].

Flow cytometric cell cycle analysis and CFSE-based proliferation tracking revealed nuanced, lineage-specific responses to PLLA-GO exposure. In both the FN1 cells and mBMSCs, the control groups exhibited a predominance of cells in the S phase, indicative of high intrinsic proliferative activity. However, exposure to PLLA-GO resulted in a marked accumulation in the G0/G1 phase and a concomitant decrease in S-phase cells—approximately 73.5% in the FN1 cells and 53.2% in the mBMSCs—suggesting a modulation of cell cycle dynamics, potentially linked to stress checkpoint activation or early-stage adaptation to the modified microenvironment. This transient arrest may reflect a shift toward a quiescent state, reducing short-term proliferative output [57,58].

A more longitudinal evaluation of proliferative behavior revealed distinct adaptation patterns. In the FN1 cells, despite G0/G1 accumulation at the analyzed time point, generation tracking showed no significant reduction in overall proliferation across time. This suggests a reversible response to initial biomaterial contact, followed by re-establishment of proliferative equilibrium, supporting the hypothesis that fibroblasts activate intrinsic adaptive mechanisms that preserve homeostatic proliferation even in a modified environment [58,59].

Regarding mesenchymal stem cells (mBMSCs), CFSE-based generation tracking revealed a transient modulation in cell division kinetics. A significant alteration was observed in second-generation progression, suggesting an initial shift in proliferative rate following contact with the PLLA-GO nanocomposite. This early modulation may reflect an adaptive phase to the interfacial microenvironment or a temporary response to biophysical and biochemical stimuli emanating from the nanocomposite surface [60,61]. The absence of persistent differences in subsequent generations indicates that mBMSCs stabilized their division kinetics, aligning with the proliferative profile of the control group. This behavior may reflect homeostatic regulatory mechanisms or the attenuation of the material-driven stimulus over time. Notably, the transient nature of this effect suggests that PLLA-GO may influence early cell cycle events without impairing the long-term proliferative capacity of mBMSCs—an essential consideration for their application in tissue engineering [62].

HUVECs displayed a more complex and nonlinear response. While cell cycle analysis showed no significant changes in phase distribution following PLLA-GO exposure (*p* ≥ 0.05), CFSE profiling revealed a multiphasic proliferation pattern characterized by accelerated entry into the second generation, an absence of detectable cells in the seventh generation, and significant accumulation in later divisions (4th and 10th generations). This profile suggests that PLLA-GO may influence HUVEC division kinetics in a discontinuous manner—potentially triggering initial proliferative activation followed by delayed progression, senescence, or selective cell loss in intermediate generations [63,64].

The pronounced presence of acellular debris in the FN1 cell cultures—exhibiting irregular, fibrous, or lamellar morphology—indicates biophysical degradation of the PLLA-GO scaffold. This phenomenon is primarily attributed to hydrolysis of the PLLA matrix in aqueous culture conditions and may be exacerbated by graphene oxide-induced alterations in surface hydrophilicity and interfacial area [65,66].

The markedly intensified degradation observed in fibroblast cultures, as compared to other lineages, suggests active cellular contributions to scaffold breakdown. One plausible mechanism involves localized acidification of the pericellular environment [67], as fibroblasts exhibit high glycolytic metabolism and secrete lactic acid [68,69]. This acidification can lower the local pH, thereby accelerating the hydrolysis of PLLA ester bonds, as supported by Silva et al. [70].

In addition, fibroblasts may contribute enzymatically, as esterases and lipases—typically linked to lipid metabolism have been implicated in the secondary hydrolytic cleavage of polymer matrices [71,72]. The proximity of these enzymes to the scaffold surface may facilitate site-specific polymer degradation.

Furthermore, the inherent contractile activity of fibroblasts [73], essential for matrix remodeling and mechanotransduction, can impose localized mechanical forces on the scaffold. Such tension may induce microfractures and structural vulnerabilities, enhancing susceptibility to hydrolysis and fragmentation. The nanostructured architecture of PLLA-GO modulated by GO incorporation may further influence cell adhesion and traction forces [74].

The absence of comparable degradation in HUVEC cultures supports the lineage-specific nature of material interaction. Endothelial cells, given their role in angiogenesis and distinct adhesion and contractile behaviors, appear to generate a less acidic and mechanically disruptive microenvironment, mitigating scaffold degradation in contrast to fibroblasts [54].

The integrated evaluation of mitochondrial electrical potential (ΔΨm) assessed by flow cytometry, combined with the inflammatory cytokine profile, offers a robust mechanistic perspective on the cellular interactions with the PLLA-GO nanocomposite.

The preservation of an active ΔΨm in the majority of the FN1 cells and mBMSCs exposed to the material suggests the maintenance of energetic homeostasis and mitochondrial integrity in these cell lines. This finding aligns with the viability observations—in terms of overall proliferative capacity over time—previously discussed for these cells. This result may indicate a lower intrinsic sensitivity of fibroblasts and mesenchymal stem cells to the direct or indirect effects of PLLA-GO regarding mitochondrial function [75].

This lower sensitivity can be attributed to several intrinsic cellular protective mechanisms. The literature suggests a higher antioxidant capacity in fibroblasts and mBMSCs, as evidenced by their metabolic plasticity and the expression of enzymes like superoxide dismutase (SOD) and catalase [76]. This could neutralize oxidative stress potentially induced by PLLA-GO, thereby protecting mitochondrial function. Additionally, the efficient regulation of mitochondrial biogenesis and dynamics, including mitophagy to remove damaged organelles, can also contribute to the resilience of these cells [77,78]. Furthermore, the activation of protective signaling pathways, such as AMPK/SIRT1, in response to stress may play a crucial role in maintaining mitochondrial homeostasis [79]. Finally, differences in mitochondrial membrane composition may influence susceptibility to material-induced damage [80]. The lower mitochondrial sensitivity observed in these lineages contrasts sharply with the response of HUVECs.

In striking contrast to the other cell lines, exposure of HUVECs to PLLA-GO resulted in a significant 32.19% reduction in active mitochondrial potential compared to the control group. This decline was accompanied by a substantial, approximately 10-fold increase over the control, in the proportion of cells exhibiting inactive mitochondrial potential. This disparity indicates a heightened sensitivity of HUVECs to the nanocomposite interface.

This atypical response raises the hypothesis that the endothelial lineage may have a limited capacity to mitigate stress induced by GO’s functional groups, or that the nature of the cell–material interaction is intrinsically more disruptive to the maintenance of these cells’ mitochondrial homeostasis. Since mitochondrial integrity plays a significant role in cellular function and persistence [80,81], the observation in HUVECs raises important questions about the potential long-term consequences of this alteration for vascular functionality and stability.

The investigation into the inflammatory cytokine profile, quantified by Cytometric Bead Array (CBA), revealed highly unique response patterns for each cell line studied. Their distinct physiological functions are essential in elucidating the complex biomaterial-cell interactions. It is important to note that the scope of cytokines quantified varied between cell lines, as the selection of CBA analysis panels was guided by optimization and specificity for the expected cytokine secretion profile of each cell type.

The basal elevation of Interleukin-6 (IL-6) levels observed in the control group fibroblasts (130.10 pg/mL, Table 5) suggests an intrinsic response to the culture microenvironment. This may influence cell cycle regulation through its pro-inflammatory action and as a growth factor, stimulating the G0/G1-S transition via activation of the Janus Kinase/Signal Transducer and Activator of Transcription 3 (JAK/STAT3) and Phosphatidylinositol-3 Kinase/Protein Kinase B (PI3K/AKT) pathways [82]. In contrast, the significant reduction in IL-6 levels detected in cells exposed to PLLA-GO—concomitant with the previously observed lower proliferation—suggests that the nanocomposite may negatively modulate the production or secretion of IL-6 by these cells. This downregulation of IL-6 could, in turn, contribute to the inhibition of downstream signaling pathways, adversely impacting progression to the S phase and, consequently, delaying or even limiting cell growth.

Regarding the inflammatory response of mBMSCs, quantitative cytokine analysis by CBA showed a statistically significant modulation (*p* = 0.0314) in Tumor Necrosis Factor alpha (TNF-α) levels, specifically in the group exposed to the PLLA-GO nanocomposite [83]. This significant elevation of TNF-α—a pleiotropic pro-inflammatory cytokine with a recognized central role in regulating intricate inflammatory, immunological, and tissue repair processes—indicates a prominent action of this material in orchestrating the inflammatory response in this cell line [84].

It is postulated that this modulation is induced by the complex biomaterial–cell interaction established between the nanocomposite and mBMSCs. Although dedicated in vitro studies involving mBMSCs and PLLA-GO are scarce, the existing literature demonstrates the intrinsic ability of graphene oxide (GO) to modulate the immune response in cell lines relevant to tissue engineering. For instance, the study by Luque-Campos et al. [83] elucidated GO’s potential to influence macrophage polarization, which can, through paracrine mechanisms, affect mBMSCs’ response. Additionally, studies evaluating scaffolds composed of GO and other polymers, such as the research by Islam et al. [85] using chitosan, corroborate GO’s ability to modulate the cell–material interface and, consequently, the cellular response. In line with these findings, the investigation conducted by Lategan et al. [84] on human BMSCs in vitro also demonstrated GO’s aptitude for modulating the immune response of these cells. This strengthens the hypothesis that the GO present in our composite significantly contributes to the elevated TNF-α levels observed in our mBMSC cultures.

Regarding the inflammatory response of the HUVEC lineage, exposure to PLLA-GO resulted in a reduction in TNF-α levels (from 97.63 pg/mL in the control to 89.11 pg/mL in the PLLA-GO group) and IL-6 levels (from 131 pg/mL in the control to 114.67 pg/mL in the PLLA-GO group). This can be attributed to the modulation of inflammatory signaling induced by the interaction of graphene oxide (GO) with the endothelial cells. The interaction of GO with the cell membrane or its internalization may inhibit the activation of key transcription factors like NF-κB, resulting in a decrease in the gene expression of these pro-inflammatory cytokines. Alternatively, this reduction might represent a later phase of the cellular response to the nanomaterial, where mechanisms of inflammatory resolution or cellular adaptation lead to the downregulation of cytokine production.

Collectively, these findings highlight the imperative for rigorous, cell-context-specific preclinical evaluation and optimized fabrication parameters to ensure the safe and effective application of PLLA-GO in regenerative strategies.

## 5. Conclusions

Our findings demonstrate that the poly(L-lactic acid) and graphene oxide (PLLA-GO) nanocomposite exhibits satisfactory primary biocompatibility with key cell types involved in regenerative processes, including human fibroblasts (FN1 cells), murine mesenchymal stem cells (mBMSCs), and human umbilical vein endothelial cells (HUVECs). However, this biocompatibility is distinctly cell type-dependent, eliciting divergent functional responses. Notably, we observed accelerated material degradation associated with fibroblast activity, transient inflammatory activation in stem cells, and pronounced mitochondrial dysfunction in endothelial cells. These outcomes underscore that PLLA-GO biocompatibility cannot be universally assumed across cell types. Therefore, rigorous, comparative, and lineage-specific preclinical evaluations are essential. The therapeutic efficacy of this nanocomposite in tissue engineering and regenerative medicine will ultimately hinge on its compatibility with the specific cellular microenvironment of the target tissue, as well as the precise optimization of its processing and formulation to ensure both functional performance and biosafety.

## Figures and Tables

**Table 1:** Specifications of cytokine kits used for quantification of inflammatory mediators by cell line.

Cell Types	Kit	Catalog Number	Measured Cytokines	Manufacturer
FN1, HUVEC	Human Th1/Th2 Cytokine Kit	551809	IL-12p70, TNF-α, IL-10, IL-6, IL-8, IL-1β, IFN-γ, MCP-1	BD^®^ Biosciences, Heidelberg, Alemanha
mBMSCs	Mouse Th1/Th2 Cytokine Kit	551287	IL-12p70, TNF-α, IL-10, IL-6, IL-8, IL-1β, IFN-γ, MCP-1	BD^®^ Biosciences, Heidelberg, Alemanha

Note—IL-12p70 refers to Interleukin-12p70; TNF-α stands for Tumor Necrosis Factor alpha; IL-10 is Interleukin-10; IL-6 denotes Interleukin-6; IL-1β represents Interleukin-1 beta; IL-8 is Interleukin-8; IFN-γ signifies Interferon Gamma; and MCP-1 corresponds to Monocyte Chemoattractant Protein-1.

**Table 2:** Cell cycle distribution in different cell lines after PLLA-GO exposure.

Cell Cycle (Phase)	FN1 vs. Control (%)	mBMSCs vs. Control (%)	HUVEC vs. Control (%)
Debris	↑ (34.38 vs. 5.93; *p* = 0.03)	↑ (34.73 vs. 5.40; *p* = 0.005)	↔ (2.8 vs. 3.8; *p* = 0.34)
G0/G1	↑ (71.74 vs. 15.27; *p* = 0.02)	↑ (61.89 vs. 39.98; *p* = 0.039)	↔ (54.26 vs. 61.93; *p* = 0.13)
S	↓ (21.58 vs. 81.50; *p* = 0.005)	↓ (29.16 vs. 54.82; *p* = 0.017)	↔ (45.74 vs. 37.99; *p* = 0.13)
G2/M	↔ (5.93 vs. 3.23; *p* = 0.28)	↔ (8.96 vs. 5.21; *p* = 0.12)	↔ (0 vs. 0.09; *p* = 0.08)

Note: ↑: Significant increase (*p* ≤ 0.05). ↓: Significant reduction (*p* ≤ 0.05). ↔: No significant change (*p* ≥ 0.05). Values expressed as percentage mean. For the FN1 cells, the control group exhibited high proliferative activity, predominantly in the S phase. However, exposure to the PLLA-GO nanocomposite significantly altered the cell cycle, leading to a notable retention of cells in the G0/G1 phase and a corresponding reduction in the S phase (Table 2, Figure 3A,B). The G2/M phase remained statistically unchanged compared to the control group (*p* = 0.28). A substantial increase in the debris fraction was also observed in the PLLA-GO group, suggesting potential material instability in the liquid medium, possibly due to hydrolytic degradation of the polymeric matrix and the subsequent release or agglomeration of graphene oxide.

**Table 3:** Concentration of mediators across generations in different cell lines after PLLA-GO exposure.

Generations	FN1 vs. Control (pg/mL)	mBMSCs vs. Control (pg/mL)	HUVEC vs. Control (pg/mL)
2	↔ (48.30 vs. 48.01; *p* = 0.89)	↑ (46.26 vs. 43.95; *p* = 0.003); Δ + 4.99%	↓ (7.8 vs. 9.24; *p* = 0.0005); Δ − 18.46%
3	↔ (31.88 vs. 31.28; *p* = 0.61)	↔ (34.03 vs. 36.34; *p* = 0.34)	↔ (9.89 vs. 9.41; *p* = 0.16)
4	↔ (19.83 vs. 20.72; *p* = 0.42)	↔ (19.71 vs. 20.21; *p* = 0.72)	↑ (16.53 vs. 8.61; *p* = 0.005); Δ + 47.91%
5	N.A	N.A	↓ (20.28 vs. 47.87; *p* = 0.003); Δ − 136.05%
6	N.A	N.A	↔ (35.13 vs. 32.05; *p* = 0.95)
7	N.A	N.A	N.D.
8	N.A	N.A	↔ (0.61 vs. 0.75; *p* = 0.65)
9	N.A	N.A	↔ (1.33 vs. 0.62; *p* = 0.23)
10	N.A	N.A	↓ (19.83 vs. 20.72; *p* < 0.0001); Δ − 4.49%

Note: ↑: Significant increase (*p* ≤ 0.05). ↓: Significant reduction (*p* ≤ 0.05). ↔: No significant change (*p* ≥ 0.05). Δ +: Percentage increase relative to control. Δ −: Percentage reduction relative to control. Values are expressed as mean. N.D.: Not detectable. N.A.: Not applicable.

**Table 4:** Changes in mitochondrial electrical potential induced by PLLA-GO in different cell lines.

ΔΨ	FN1 vs. Control (%)	mBMSCs vs. Control (%)	HUVEC vs. Control (%)
Active cells	↔ (88.58 vs. 90.76)	↔ (91.6 vs. 93.03)	↓ (65.82 vs. 97.07); Δ − 32.19%
Inactive cells	↔ (11.56 vs. 9.32)	↔ (8.42 vs. 7.03)	↑ (34.24 vs. 3.04); Δ + 1026.32%

Note: ↑: Significant increase (*p* ≤ 0.05). ↓: Significant reduction (*p* ≤ 0.05). ↔: No significant change (*p* ≥ 0.05). Δ +: Percentage increase relative to control. Δ −: Percentage reduction relative to control. Values are expressed as the mean.

**Table 5:** Modulation of inflammatory cytokine concentration induced by PLLA-GO in different cell lines.

Citokines	FN1 vs. Control (pg/mL)	mBMSCs vs. Control (pg/mL)	HUVEC vs. Control (pg/mL)
IL-12p70	↔ (73.14 vs. 76.33; *p* = 0.68)	↔ (150.52 vs. 165.43; *p* = 0.39)	↔ (73.13 vs. 76.33; *p* = 0.68)
TNF-α	↔ (89.11 vs. 97.63; *p* = 0.052)	↑ (179.67 vs. 147.68; *p* = 0.03); Δ + 21.66%	↓ (89.11 vs. 97.63; *p* = 0.05); Δ − 8.73%
IL-10	↔ (96.21 vs. 99.75; *p* = 0.39)	↔ (221.52 vs. 224.36; *p* = 0.71)	↔ (96.21 vs. 99.76; *p* = 0.39)
IL-6	↓ (114.67 vs. 130.10; *p* = 0.019); Δ − 1.86%	↔ (402.57 vs. 388.37; *p* = 0.62)	↓ (114.67 vs. 131.00; *p* = 0.02); Δ − 12.47%
IL-8	↔ (150.00 vs. 155.20; *p* = 0.42)	↔ (185.20 vs. 190.50; *p* = 0.55)	↔ (165.00 vs. 170.10; *p* = 0.38)
1L-1β	↔ (301.40 vs. 319.25; *p* = 0.34)	↔ (330.10 vs. 335.70; *p* = 0.60)	↔ (310.00 vs. 315.30; *p* = 0.47)
IFN-γ	↔ (155.00 vs. 158.50; *p* = 0.68)	↔ (157.62 vs. 164.72; *p* = 0.1)	↔ (148.70 vs. 150.10; *p* = 0.82)
MCP-1	↔ (7.500 vs. 7.700; *p* = 0.52)	↔ (8.675. vs. 8.076; *p* = 0.23)	↔ (7.050 vs. 7.200; *p* = 0.70)

Note: ↑: Significant increase (*p* ≤ 0.05). ↓: Significant reduction (*p* ≤ 0.05). ↔: No significant change (*p* ≥ 0.05). Δ +: Percentage increase relative to control. Δ −: Percentage reduction relative to control. Values are expressed as mean. N.A.: Not analyzed.

## Data Availability

The authors confirm that the data supporting the findings of this study are available within the article and its Supplementary Materials. Data are also available on request from the authors.

## Supplementary Materials

The following supporting information can be downloaded at: https://www.mdpi.com/article/10.3390/pharmaceutics17070892/s1, Figure S1: PLLA-GO Nanocomposite Fabrication and Quality Control Steps; Figure S2: Steps for Metallization of PLLA-GO Nanocomposite Samples.

## References

[1] Ferraz M.P. (2024). An Overview on the Big Players in Bone Tissue Engineering: Biomaterials, Scaffolds and Cells. Int. J. Mol. Sci..

[2] Farjaminejad S., Farjaminejad R., Hasani M., Garcia-Godoy F., Abdouss M., Marya A., Harsoputranto A., Jamilian A. (2024). Advances and Challenges in Polymer-Based Scaffolds for Bone Tissue Engineering: A Path Towards Personalized Regenerative Medicine. Polymers.

[3] Muzzio N., Moya S., Romero G. (2021). Multifunctional Scaffolds and Synergistic Strategies in Tissue Engineering and Regenerative Medicine. Pharmaceutics.

[4] Khan A.R., Gholap A.D., Grewal N.S., Jun Z., Khalid M., Zhang H.-J. (2025). Advances in Smart Hybrid Scaffolds: A Strategic Approach for Regenerative Clinical Applications. Eng. Regen..

[5] Mohammadi Nasr S., Rabiee N., Hajebi S., Ahmadi S., Fatahi Y., Hosseini M., Bagherzadeh M., Ghadiri A.M., Rabiee M., Jajarmi V. (2020). Biodegradable Nanopolymers in Cardiac Tissue Engineering: From Concept Towards Nanomedicine. Int. J. Nanomed..

[6] Gunatillake P. (2003). Biodegradable Synthetic Polymers for Tissue Engineering. Eur. Cell Mater..

[7] Khouri N.G., Bahú J.O., Blanco-Llamero C., Severino P., Concha V.O.C., Souto E.B. (2024). Polylactic Acid (PLA): Properties, Synthesis, and Biomedical Applications—A Review of the Literature. J. Mol. Struct..

[8] Limsukon W., Rubino M., Rabnawaz M., Lim L.-T., Auras R. (2023). Hydrolytic Degradation of Poly(Lactic Acid): Unraveling Correlations between Temperature and the Three Phase Structures. Polym. Degrad. Stab..

[9] Quirk R.A., Davies M.C., Tendler S.J.B., Chan W.C., Shakesheff K.M. (2001). Controlling Biological Interactions with Poly(Lactic Acid) by Surface Entrapment Modification. Langmuir.

[10] da Silva D., Kaduri M., Poley M., Adir O., Krinsky N., Shainsky-Roitman J., Schroeder A. (2018). Biocompatibility, Biodegradation and Excretion of Polylactic Acid (PLA) in Medical Implants and Theranostic Systems. Chem. Eng. J..

[11] Maestrelli L.M.D., Oyama H.T.T., Muñoz P.A.R., Cestari I.A., Fechine G.J.M. (2021). Role of Graphene Oxide on the Mechanical Behaviour of Polycarbonate-Urethane/Graphene Oxide Composites. Mater. Res..

[12] Viprya P., Kumar D., Kowshik S. Study of Different Properties of Graphene Oxide (GO) and Reduced Graphene Oxide (RGO). Proceedings of the RAiSE-2023.

[13] Arriagada P., Palza H., Palma P., Flores M., Caviedes P. (2018). Poly(Lactic Acid) Composites Based on Graphene Oxide Particles with Antibacterial Behavior Enhanced by Electrical Stimulus and Biocompatibility. J. Biomed. Mater. Res. A.

[14] Zhou B., Zheng C., Zhang R., Xue S., Zheng B., Shen H., Sheng Y., Zhang H. (2024). Graphene Oxide-Enhanced and Dynamically Crosslinked Bio-Elastomer for Poly(Lactic Acid) Modification. Molecules.

[15] Dey K., Roca E., Ramorino G., Sartore L. (2020). Progress in the Mechanical Modulation of Cell Functions in Tissue Engineering. Biomater. Sci..

[16] Da Silva K., Kumar P., Choonara Y.E. (2025). The Paradigm of Stem Cell Secretome in Tissue Repair and Regeneration: Present and Future Perspectives. Wound Repair. Regen..

[17] Park J.Y.C., King A., Björk V., English B.W., Fedintsev A., Ewald C.Y. (2023). Strategic Outline of Interventions Targeting Extracellular Matrix for Promoting Healthy Longevity. Am. J. Physiol. Cell Physiol..

[18] Tanaka K., Ogino R., Yamakawa S., Suda S., Hayashida K. (2022). Role and Function of Mesenchymal Stem Cells on Fibroblast in Cutaneous Wound Healing. Biomedicines.

[19] Schuster R., Rockel J.S., Kapoor M., Hinz B. (2021). The Inflammatory Speech of Fibroblasts. Immunol. Rev..

[20] Nancarrow-Lei R., Mafi P., Mafi R., Khan W. (2017). A Systemic Review of Adult Mesenchymal Stem Cell Sources and Their Multilineage Differentiation Potential Relevant to Musculoskeletal Tissue Repair and Regeneration. Curr. Stem Cell Res. Ther..

[21] Maqsood M., Kang M., Wu X., Chen J., Teng L., Qiu L. (2020). Adult Mesenchymal Stem Cells and Their Exosomes: Sources, Characteristics, and Application in Regenerative Medicine. Life Sci..

[22] Han Y., Yang J., Fang J., Zhou Y., Candi E., Wang J., Hua D., Shao C., Shi Y. (2022). The Secretion Profile of Mesenchymal Stem Cells and Potential Applications in Treating Human Diseases. Signal Transduct. Target. Ther..

[23] Xue Z., Liao Y., Li Y. (2024). Effects of Microenvironment and Biological Behavior on the Paracrine Function of Stem Cells. Genes Dis..

[24] Rouwkema J., Khademhosseini A. (2016). Vascularization and Angiogenesis in Tissue Engineering: Beyond Creating Static Networks. Trends Biotechnol..

[25] Ren B., Jiang Z., Murfee W.L., Katz A.J., Siemann D., Huang Y. (2023). Realizations of Vascularized Tissues: From in Vitro Platforms to in Vivo Grafts. Biophys. Rev..

[26] Chen J., Zhang D., Wu L.-P., Zhao M. (2023). Current Strategies for Engineered Vascular Grafts and Vascularized Tissue Engineering. Polymers.

[27] Hummers W.S., Offeman R.E. (1958). Preparation of Graphitic Oxide. J. Am. Chem. Soc..

[28] Laraba S.R., Ullah N., Bouamer A., Ullah A., Aziz T., Luo W., Djerir W., Zahra Q.U.A., Rezzoug A., Wei J. (2023). Enhancing Structural and Thermal Properties of Poly(Lactic Acid) Using Graphene Oxide Filler and Anionic Surfactant Treatment. Molecules.

[29] Azizli M.J., Honarkar H., Vafa E., Parham S., Rezaeeparto K., Azizli F., Kianfar M.R., Zarei M.B., Amani A.M., Mokhtary M. (2024). Synthesis and Characterization of the Novel Nanocomposites Based on Graphene Oxide/PLLA/PEG-PPG/PLCL Hybrids for Mechanical and Biomedical Applications. J. Polym. Environ..

[30] Qiu Z., Lin X., Zou L., Fu W., Lv H. (2024). Effect of Graphene Oxide/Poly-L-Lactic Acid Composite Scaffold on the Biological Properties of Human Dental Pulp Stem Cells. BMC Oral Health.

[31] Bouider B., Bouakaz B.S., Haffad S., Berrayah A., Magueresse A., Grohens Y., Habi A. (2023). Composite Nanoarchitectonics of Poly(Lactic Acid)/Metal Organic Framework with Property Investigations Toward Packaging Applications. J. Inorg. Organomet. Polym. Mater..

[32] Silva T.S., Soares M.M., Carreira A.C.O., Matias G.d.S.S., Tegon C.C., Massi M., Oliveira A.d.A., Júnior L.N.d.S., de Carvalho H.J.C., Almeida G.H.D.R. (2021). Biological Characterization of Polymeric Matrix and Graphene Oxide Biocomposites Filaments for Biomedical Implant Applications: A Preliminary Report. Polymers.

[33] Wang H., Qiu Z. (2012). Crystallization Kinetics and Morphology of Biodegradable Poly(l-Lactic Acid)/Graphene Oxide Nanocomposites: Influences of Graphene Oxide Loading and Crystallization Temperature. Thermochim. Acta.

[34] Yao X., Yan Z., Wang X., Jiang H., Qian Y., Fan C. (2021). The Influence of Reduced Graphene Oxide on Stem Cells: A Perspective in Peripheral Nerve Regeneration. Regen. Biomater..

[35] Luo Y., Shen H., Fang Y., Cao Y., Huang J., Zhang M., Dai J., Shi X., Zhang Z. (2015). Enhanced Proliferation and Osteogenic Differentiation of Mesenchymal Stem Cells on Graphene Oxide-Incorporated Electrospun Poly(Lactic-Co-Glycolic Acid) Nanofibrous Mats. ACS Appl. Mater. Interfaces.

[36] Kim M., Jeong J.H., Lee J.-Y., Capasso A., Bonaccorso F., Kang S.-H., Lee Y.-K., Lee G.-H. (2019). Electrically Conducting and Mechanically Strong Graphene–Polylactic Acid Composites for 3D Printing. ACS Appl. Mater. Interfaces.

[37] González-Rodríguez L., Pérez-Davila S., Lama R., López-Álvarez M., Serra J., Novoa B., Figueras A., González P. (2023). 3D Printing of PLA:CaP:GO Scaffolds for Bone Tissue Applications. RSC Adv..

[38] Oktay B., Özerol E.A., Sahin A., Gunduz O., Ustundag C.B. (2022). Production and Characterization of PLA/HA/GO Nanocomposite Scaffold. ChemistrySelect.

[39] Choi H.W., Cox A., Mofarah H.M., Jabbour G. (2024). Mechanical and Electrical Properties of 3D-Printed Highly Conductive Reduced Graphene Oxide/Polylactic Acid Composite. Adv. Eng. Mater..

[40] Pandey A., Singh J., Singh M., Singh G., Parmar A.S., Chaudhary S. (2025). Graphene Oxide/Polylactic Acid Composites with Enhanced Electrical and Mechanical Properties for 3D-Printing Materials. J. Mol. Struct..

[41] Guo W., Yang Y., Liu C., Bu W., Guo F., Li J., Wang E., Peng Z., Mai H., You H. (2023). 3D Printed TPMS Structural PLA/GO Scaffold: Process Parameter Optimization, Porous Structure, Mechanical and Biological Properties. J. Mech. Behav. Biomed. Mater..

[42] da Silva T.S., Horvath-Pereira B.d.O., da Silva-Júnior L.N., Fireman J.V.B.T., Mattar M., Félix M., Buchaim R.L., Carreira A.C.O., Miglino M.A., Soares M.M. (2023). Three-Dimensional Printing of Graphene Oxide/Poly-L-Lactic Acid Scaffolds Using Fischer–Koch Modeling. Polymers.

[43] He Y., Yan J., He X., Weng W., Cheng K. (2022). PLLA/Graphene Nanocomposites Membranes with Improved Biocompatibility and Mechanical Properties. Coatings.

[44] Qiu Z., Guan W. (2014). In Situ Ring-Opening Polymerization of Poly(l-Lactide)-Graft-Graphene Oxide and Its Effect on the Crystallization Kinetics and Morphology of Biodegradable Poly(l-Lactide) at Low Loadings. RSC Adv..

[45] Bayer I. (2017). Thermomechanical Properties of Polylactic Acid-Graphene Composites: A State-of-the-Art Review for Biomedical Applications. Materials.

[46] Souza D.H.S., Santoro P.V., Dias M.L. (2017). Isothermal Crystallization Kinetics of Poly(Lactic Acid) Stereocomplex/Graphene Nanocomposites. Mater. Res..

[47] Winn S.R., Schmitt J.M., Buck D., Hu Y., Grainger D., Hollinger J.O. (1999). Tissue-Engineered Bone Biomimetic to Regenerate Calvarial Critical- Sized Defects in Athymic Rats. J. Biomed. Mater. Res..

[48] Pinto A.M., Moreira S., Gonçalves I.C., Gama F.M., Mendes A.M., Magalhães F.D. (2013). Biocompatibility of Poly(Lactic Acid) with Incorporated Graphene-Based Materials. Colloids Surf. B Biointerfaces.

[49] Krausgruber T., Fortelny N., Fife-Gernedl V., Senekowitsch M., Schuster L.C., Lercher A., Nemc A., Schmidl C., Rendeiro A.F., Bergthaler A. (2020). Structural Cells Are Key Regulators of Organ-Specific Immune Responses. Nature.

[50] You D., Li K., Guo W., Zhao G., Fu C. (2019). Poly(Lactic-Co-Glycolic Acid)/Graphene Oxide Composites Combined with Electrical Stimulation in Wound Healing: Preparation and Characterization. Int. J. Nanomed..

[51] Hennigs J.K., Matuszcak C., Trepel M., Körbelin J. (2021). Vascular Endothelial Cells: Heterogeneity and Targeting Approaches. Cells.

[52] Su J., Song Y., Zhu Z., Huang X., Fan J., Qiao J., Mao F. (2024). Cell–Cell Communication: New Insights and Clinical Implications. Signal Transduct. Target. Ther..

[53] Sulaksono H., Annisa A., Ruslami R., Mufeeduzzaman M., Panatarani C., Hermawan W., Ekawardhani S., Joni I.M. (2024). Recent Advances in Graphene Oxide-Based on Organoid Culture as Disease Model and Cell Behavior—A Systematic Literature Review. Int. J. Nanomed..

[54] Zhang X., Zhang S., Wang T. (2022). How the Mechanical Microenvironment of Stem Cell Growth Affects Their Differentiation: A Review. Stem Cell Res. Ther..

[55] AbouAitah K., Sabbagh F., Kim B.S. (2023). Graphene Oxide Nanostructures as Nanoplatforms for Delivering Natural Therapeutic Agents: Applications in Cancer Treatment, Bacterial Infections, and Bone Regeneration Medicine. Nanomaterials.

[56] Hu X., Li J., Fu M., Zhao X., Wang W. (2021). The JAK/STAT Signaling Pathway: From Bench to Clinic. Signal Transduct. Target. Ther..

[57] Wang X., Wang H., Jiang K., Zhang Y., Zhan C., Ying M., Zhang M., Lu L., Wang R., Wang S. (2019). Liposomes with Cyclic RGD Peptide Motif Triggers Acute Immune Response in Mice. J. Control. Release.

[58] Hao Q., Zong X., Sun Q., Lin Y.-C., Song Y.J., Hashemikhabir S., Hsu R.Y., Kamran M., Chaudhary R., Tripathi V. (2020). The S-Phase-Induced LncRNA SUNO1 Promotes Cell Proliferation by Controlling YAP1/Hippo Signaling Pathway. Elife.

[59] Brink T.T., Damanik F., Rotmans J.I., Moroni L. (2024). Unraveling and Harnessing the Immune Response at the Cell–Biomaterial Interface for Tissue Engineering Purposes. Adv. Heal Mater..

[60] Ma Q., Wang X., Feng B., Liang C., Wan X., El-Newehy M., Abdulhameed M.M., Mo X., Wu J. (2024). Fiber Configuration Determines Foreign Body Response of Electrospun Scaffolds: In Vitro and in Vivo Assessments. Biomed. Mater..

[61] Ghosh K., Pan Z., Guan E., Ge S., Liu Y., Nakamura T., Ren X.-D., Rafailovich M., Clark R.A.F. (2007). Cell Adaptation to a Physiologically Relevant ECM Mimic with Different Viscoelastic Properties. Biomaterials.

[62] Dudaryeva O.Y., Bernhard S., Tibbitt M.W., Labouesse C. (2023). Implications of Cellular Mechanical Memory in Bioengineering. ACS Biomater. Sci. Eng..

[63] Chavkin N.W., Genet G., Poulet M., Jeffery E.D., Marziano C., Genet N., Vasavada H., Nelson E.A., Acharya B.R., Kour A. (2022). Endothelial Cell Cycle State Determines Propensity for Arterial-Venous Fate. Nat. Commun..

[64] Shin Y.J., Lee J.H. (2024). Exploring the Molecular and Developmental Dynamics of Endothelial Cell Differentiation. Int. J. Stem Cells.

[65] Bikiaris N.D., Koumentakou I., Samiotaki C., Meimaroglou D., Varytimidou D., Karatza A., Kalantzis Z., Roussou M., Bikiaris R.D., Papageorgiou G.Z. (2023). Recent Advances in the Investigation of Poly(Lactic Acid) (PLA) Nanocomposites: Incorporation of Various Nanofillers and Their Properties and Applications. Polymers.

[66] Castro-Aguirre E., Iñiguez-Franco F., Samsudin H., Fang X., Auras R. (2016). Poly(Lactic Acid)—Mass Production, Processing, Industrial Applications, and End of Life. Adv. Drug Deliv. Rev..

[67] Park S.A., Lee S.J., Seok J.M., Lee J.H., Kim W.D., Kwon I.K. (2018). Fabrication of 3D Printed PCL/PEG Polyblend Scaffold Using Rapid Prototyping System for Bone Tissue Engineering Application. J. Bionic Eng..

[68] Lemons J.M.S., Feng X.-J., Bennett B.D., Legesse-Miller A., Johnson E.L., Raitman I., Pollina E.A., Rabitz H.A., Rabinowitz J.D., Coller H.A. (2010). Quiescent Fibroblasts Exhibit High Metabolic Activity. PLoS Biol..

[69] Vander Heiden M.G., Cantley L.C., Thompson C.B. (2009). Understanding the Warburg Effect: The Metabolic Requirements of Cell Proliferation. Science.

[70] Silva R.R.A., Marques C.S., Arruda T.R., Teixeira S.C., de Oliveira T.V. (2023). Biodegradation of Polymers: Stages, Measurement, Standards and Prospects. Macromol.

[71] Kürten C., Carlberg B., Syrén P.-O. (2016). Mechanism-Guided Discovery of an Esterase Scaffold with Promiscuous Amidase Activity. Catalysts.

[72] Tokiwa Y., Suzuki T. (1977). Hydrolysis of Polyesters by Lipases. Nature.

[73] Shvetsova E.V., Rogovaya O.S., Tkachenko S.B., Kiselev I.V., Vasil’ev A.V., Terskikh V.V. (2008). Contractile Capacity of Fibroblasts from Different Sources in the Model of Living Skin Equivalent. Biol. Bull..

[74] Naganuma T. (2017). The Relationship between Cell Adhesion Force Activation on Nano/Micro-Topographical Surfaces and Temporal Dependence of Cell Morphology. Nanoscale.

[75] Cun X., Hosta-Rigau L. (2020). Topography: A Biophysical Approach to Direct the Fate of Mesenchymal Stem Cells in Tissue Engineering Applications. Nanomaterials.

[76] Sahlender B., Windolf J., Suschek C.V. (2022). Superoxide Dismutase and Catalase Significantly Improve the Osteogenic Differentiation Potential of Osteogenetically Compromised Human Adipose Tissue-Derived Stromal Cells in Vitro. Stem Cell Res..

[77] Yao Y., Zhang T., Tang M. (2024). Toxicity Mechanism of Engineered Nanomaterials: Focus on Mitochondria. Environ. Pollut..

[78] Salisbury R.L., Agans R., Huddleston M.E., Snyder A., Mendlein A., Hussain S. (2018). Toxicological Mechanisms of Engineered Nanomaterials: Role of Material Properties in Inducing Different Biological Responses. Handbook of Developmental Neurotoxicology.

[79] Herzig S., Shaw R.J. (2018). AMPK: Guardian of Metabolism and Mitochondrial Homeostasis. Nat. Rev. Mol. Cell Biol..

[80] Sharma P., Sampath H. (2019). Mitochondrial DNA Integrity: Role in Health and Disease. Cells.

[81] Chen W., Zhao H., Li Y. (2023). Mitochondrial Dynamics in Health and Disease: Mechanisms and Potential Targets. Signal Transduct. Target. Ther..

[82] Abaurrea A., Araujo A.M., Caffarel M.M. (2021). The Role of the IL-6 Cytokine Family in Epithelial–Mesenchymal Plasticity in Cancer Progression. Int. J. Mol. Sci..

[83] Luque-Campos N., Bustamante-Barrientos F.A., Pradenas C., García C., Araya M.J., Bohaud C., Contreras-López R., Elizondo-Vega R., Djouad F., Luz-Crawford P. (2021). The Macrophage Response Is Driven by Mesenchymal Stem Cell-Mediated Metabolic Reprogramming. Front. Immunol..

[84] Lategan K., Alghadi H., Bayati M., De Cortalezzi M., Pool E. (2018). Effects of Graphene Oxide Nanoparticles on the Immune System Biomarkers Produced by RAW 264.7 and Human Whole Blood Cell Cultures. Nanomaterials.

[85] Islam M., Shahruzzaman, Biswas S., Sakib N., Rashid T.U. (2020). Chitosan Based Bioactive Materials in Tissue Engineering Applications—A Review. Bioact. Mater..

