# Supplementary material for: Impact of Poly(Lactic Acid) and Graphene Oxide Nanocomposite on Cellular Viability and Proliferation
Source: Pharmaceutics. 2025 Jul 9;17(7):892. doi: 10.3390/pharmaceutics17070892 (PMC12300299; doi:10.3390/pharmaceutics17070892)
Supplement: Supplementary file 1 [file pharmaceutics-17-00892-s001.zip › pharmaceutics-3708371-supplementary.pdf]

pharmaceutics

MDPI

SUPPLEMENTARY MATERIALS

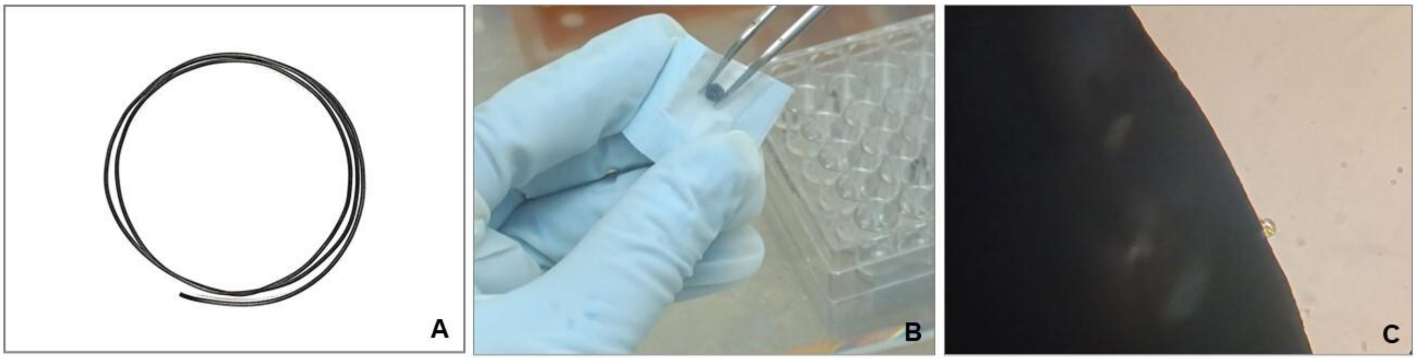

Figure S1: PLLA-GO Nanocomposite Fabrication and Quality Control Steps. This section details the manufacturing steps and quality control for the PLLA-GO nanocomposite: Polymeric PLLA-GO nanocomposite filaments were used as raw material for 3D printing discoidal scaffolds (A). The manipulation and positioning process of the scaffolds in the culture plate after nanocomposite fabrication (B). A microscopic image of PLLA-GO scaffolds cultured without cells, performed under the same experimental conditions, confirming the absence of contamination (C).

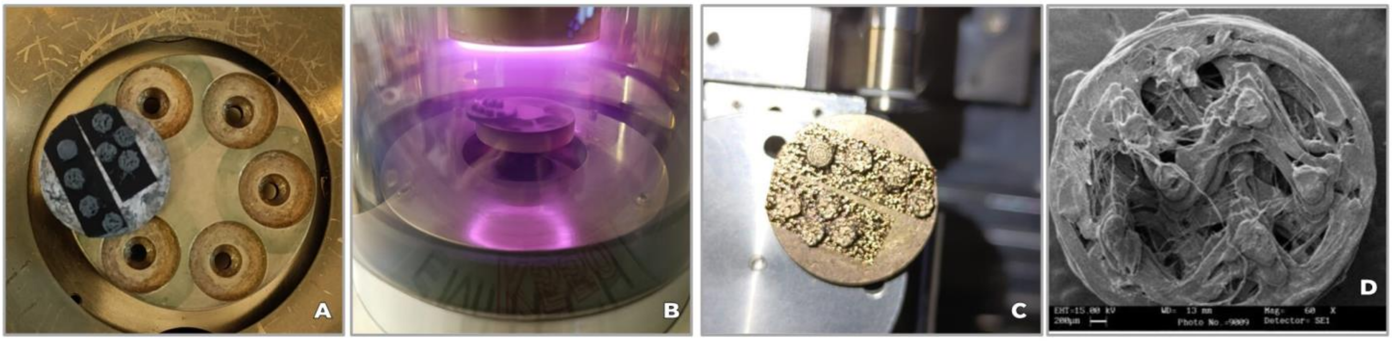

Figure S2: Steps for Metallization of PLLA-GO Nanocomposite Samples. This section details the metallization process for the PLLA-GO nanocomposite samples: (A) Positioning of the PLLA-GO nanocomposite samples on the sample holder inside the vacuum chamber of the Sputter Coater. (B) Sputtering process in progress, showing the luminous plasma (ionized argon) generated to bombard the metallic target and eject metal atoms. (C) Metallized samples removed from the Sputter Coater. A thin conductive layer of gold metal was deposited on the surface of the samples. (D) SEM images of PLLA coated with GO.
